# Integrated fetal testicular transcriptomic and epigenomic profiles during maternal nutrient restriction with dietary melatonin intervention

**DOI:** 10.1093/jas/skaf455

**Published:** 2026-01-09

**Authors:** Hala El Daous, Brittni P Littlejohn, Zully E Contreras-Correa, Shiveeli Rajput, Darcie R Sidelinger, E Heath King, Mark A Arick, Caleb O Lemley

**Affiliations:** Department of Animal and Dairy Sciences, Mississippi State University, Mississippi State, MS, 39762; Faculty of Veterinary Medicine, Benha University, Mushtuhur, Qalioubia, 13736, Egypt; Department of Animal Science, Division of Agriculture, University of Arkansas, Fayetteville, AR, 72701; Department of Animal Science, Tarleton State University, Stephenville, TX, 76402; Department of Animal and Dairy Sciences, Mississippi State University, Mississippi State, MS, 39762; Department of Pathobiology and Population Medicine, Mississippi State University College of Veterinary Medicine, Mississippi State, MS, 39762; Department of Pathobiology and Population Medicine, Mississippi State University College of Veterinary Medicine, Mississippi State, MS, 39762; Institute for Genomics, Biocomputing and Biotechnology, Mississippi State University, Mississippi State, MS, 39762; Department of Animal and Dairy Sciences, Mississippi State University, Mississippi State, MS, 39762

**Keywords:** melatonin, nutrient restriction, testicular DNA methylation, testicular transcriptomics

## Abstract

Fetal development is a critical period that establishes reproductive efficiency and herd performance depending on in-utero epigenetic modifications. Dietary restrictions may affect fetal testis development and the offspring fertility. Several studies have connected genetic instability to circadian cycle disruptions, including epigenetic modifications to melatonin, a key regulator. On day 160 of gestation, 17 male-bearing Brangus heifers were assigned to one of four groups in a 2 × 2 factorial treatment arrangement: adequately fed (ADQ; 100% NRC recommendation, n = 3), nutrient restricted (RES; 60% NRC recommendation, *n* = 5), or ADQ or RES supplemented with 20 mg/d melatonin (ADQ-MEL, *n* = 5; RES-MEL, *n* = 4). On day 240 of gestation, heifers underwent Cesarean sections to collect fetuses and testicular tissues. The fetal testicular tissue was processed and analyzed using the Methyl-MiniSeq Service: Genome-wide bisulfite sequencing (Methyl MiniSeq-GWBS). Sequence reads from Methyl Mini-Seq libraries were identified using standard Illumina platform calling software for methylome profile. RNA-Seq libraries were then sequenced on the Illumina platform for transcriptome profile. The common genes between differentially methylated regions (DMRs) and differentially expressed genes (DEGs) across different treatment groups were identified by an overlap analysis using bedtools v2.31.1. There were 413 DMRs in RES-CON and ADQ-CON testicular tissues, without differential gene expression. Compared with the ADQ-CON group, the ADQ-MEL group showed 411 DMRs and a higher *KYAT1* gene expression (*P*-adj <0.05) without methylation changes. Comparing RES-MEL with RES-CON showed that 9 genes (*DAAM1*, *COL28A1*, *RPL10*, *TRPM3*, *SLIT*, *ARHGEF40*, *SYT1*, *TMEM35B*, *CSPG4B*) were expressed more in the former (*P*-adj <0.05). The only hypomethylated gene was *DAAM1* located on chromosome 10. However, 13 genes (*PTPRU*, *snRNP-E*, *TMEM59L*, *MUC5B*, *ANAPC15*, *FAM221A*, *SHCBPiL*, *PAQR5*, *PPP4R3C*, *DTNB*, *LncRNA*, *SHANK2*, *RIC3*) showed increased expression in RES-CON vs. RES-MEL without differential methylation alterations, yet there were 370 DMRs. Five genes showed increased expression in RES-MEL compared with ADQ-MEL (*P*-adj <0.05), including histone *H2B* on chromosome 23. Two genes (*PTPRU*, *TDRD10*) showed increased expression in ADQ-MEL compared with RES-MEL (*P*-adj <0.05) without affecting methylation and 344 DMRs. In conclusion, dietary melatonin supplementation to nutrient restricted dams may influence fetal development as epigenomic and transcriptomic regulators are altered.

## Teaser

Epigenetics offers a new way to study embryonic development, as it is affected by environmental influences and the causes of illness. Understanding how dietary limitation and/or melatonin supplementation alter epigenetic modifications would help us optimize pregnant cow management and male offspring development through nutritional manipulation, offering a physiological alternative to pharmacotherapies.

## Introduction

The fetal programming hypothesis suggests that stimuli that occurs during a sensitive period of development can program fetal tissues and exert organizational effects that continue throughout the offspring’s life (Vonnahme and Lemley, 2011; Lemley, 2020). It is increasingly apparent that environmental alterations, including nutritional modifications, can lead to variations in offspring growth, metabolism, reproduction, and health in later life (Caton et al., 2019). The nutritional status of the dam is crucial throughout pregnancy, as external stimuli or perturbations during gestation may result in phenotypic and metabolic alterations in the growing fetus (Reynolds et al., 2019). Beef cattle, when reared on pasture, are particularly vulnerable to nutritional deficiencies caused by seasonal variations in forage quality. Numerous studies have demonstrated the impact of maternal diet on the development of muscle and adipose tissue (Costa et al., 2021; Polizel et al., 2021; Zhang et al., 2021), and body weight (Long et al., 2021); however, research concerning the influence of maternal nutrition on the performance, metabolism, and health of male replacement animals is scarce. Nutrient restriction throughout testis development in utero may affect testis function and subsequently contribute to subfertility in mature males (McCoski et al., 2021).

Epigenetic modifications that result from alternations in prenatal nutrition during crucial developmental periods may permanently alter physiology and metabolism (Godfrey and Barker, 2001; Yan et al., 2013). Maternal nutrition affects fetal development through modifying epigenetic markers that regulate gene expression without changing DNA sequence (Lan et al., 2013). These epigenetic markers include DNA methylation, histone changes, and non-coding RNA activity (Jaenisch and Bird, 2003; Ji et al., 2016). DNA methylation regulates transcription, genomic imprinting, and X chromosome inactivation in mammals (Suzuki and Bird, 2008; Pelizzola and Ecker, 2011). Changes in maternal nutrient availability can affect fetal genome methylation by altering DNA methylation enzymes or by modifying substrate availability (Choi and Friso, 2010; Ji et al., 2016).

Melatonin has recently emerged as a potential therapeutic in alleviating adverse environmental effects such as maternal undernutrition in both sheep and cattle (Lemley et al., 2012, 2013; Contreras-Correa et al., 2021, 2022). Melatonin is known as a powerful antioxidant and blood flow regulator that can pass through the placenta into ovine and bovine fetal circulation (Lemley et al., 2012; Brockus et al., 2016). Interestingly, Contreras-Correa et al. (2021) reported that nutrient restriction decreased uterine blood flow (UBF) in pregnant Brangus heifers and melatonin supplementation increased UBF depending on the season. Melatonin has also been shown to reduce severe DNA damage, a consequence of unstable oxygen-based reactants in rats and mice models (Reiter et al., 2004) and can serve as an epigenetic modifier in human (Korkmaz and Reiter, 2008). In addition to the positive effects melatonin has on female reproductive performance, melatonin may also be a beneficial modulator of male reproductive efficiency. McCarty et al. (2018) reported increased scrotal circumference and testicular weight at the time of weaning in bull calves born from melatonin supplemented beef cattle (two subdermal ear implants containing 24 mg of melatonin) at day 180, 210, and 240 of gestation. Melatonin receptors have been found in Sertoli and Leydig cells in the testes (Frungieri et al., 2017; González-Arto et al., 2017), and exogenous melatonin upregulates gene expression of spermatogenesis-related genes (Yang et al., 2014). Despite evidence that melatonin may affect male offspring performance, the combined effects of melatonin supplementation and nutrient restriction on testicular DNA methylation and gene expression related to testis development, spermatogenesis, and fertility are still poorly understood.

We hypothesized that melatonin supplementation and a nutritional deficit throughout mid-to-late gestation would affect fetal testis DNA methylation and thereby transcriptomic profiles. We aimed to determine the differentially methylated regions (DMRs) in fetal testis tissue from nutritionally restricted or adequately fed dams that had been supplemented with melatonin or a vehicle control. Additionally, the association between transcriptome patterns and DNA methylation signatures was examined to investigate possible outcomes related to bull reproductive efficiency.

## Materials and Methods

### Animal procedure and tissue collection

The Institutional Animal Care and Use Committee at Mississippi State University approved the use and care of the study animals (#17-709). All animal breeding, diets, treatments, and Caesarean sections procedures were previously described by Contreras-Correa et al. (2021). In summary, this study included only male-bearing, spring calving Brangus heifers (*n* = 17) [ADQ-CON (*n* = 3), RES-CON (*n* = 5), ADQ-MEL (*n* = 5), and RES-MEL (*n* = 4)], that were bred to a single sire by artificial insemination. The heifers were anticipated to give birth in January 2020. In a 2 × 2 factorial design, heifers were stratified by body weight at day 160 of gestation and randomly assigned to one of four dietary treatments: adequately fed (ADQ-CON; 100% NRC recommendation), nutrient restricted (RES-CON, 60% NRC recommendation), or adequately fed or nutrient restricted supplemented with 20 mg/d of melatonin (ADQ-MEL; RES-MEL), or adequately fed (ADQ-CON; 100% NRC recommendation) (NRC, 2000). Melatonin (#14427; Cayman Chemical Company, Ann Arbor, MI, USA) feeding and dietary composition have already been discussed (Contreras-Correa et al., 2021), animals were supplemented with 20 mg/d of melatonin dissolved in 2 mL of absolute ethanol, while plain absolute ethanol served as vehicle control (CON). The heifers were fed a grain mix top dressed in the treatment CON or MEL at 0900 h, and after consuming their treatments, they were given a total mixed ration ADQ or RES using the Calan gates electronic feeding system. Weekly dietary adjustments were made in accordance with the dam’s body weight.

### Cesarean section and fetal tissue collection

According to Contreras-Correa et al. (2021) heifers were subjected to Cesarean procedures on day 240 of gestation to collect fetal tissue. A Silencer hydraulic squeeze chute was used to restrain the animals during surgeries at the H. H. Leveck Animal Research Center. Aseptic surgery was performed on the skin surrounding the incision site after a paravertebral or inverted-L block with 2% lidocaine. An incision of 20 cm was performed ventral to the paralumbar fossa’s transverse processes. The gravid uterus was delivered to the incision site using the fetus’s frontal limbs, which were identified and employed as a handle. The umbilical cord was identified, clamped, and cut following a uterine incision. For 4 wk following surgery, a veterinarian checked the cattle every day for clinical indications of infection. Following fetal extraction, testicular parenchymal tissue was harvested, deposited in cryogenic tubes, rapidly frozen in liquid nitrogen, and preserved at −80°C for subsequent analysis. Fetal testicular parenchymal tissue was sent to Zymo Research Corp (Zymo Research, Irvine, CA) for the investigation of DNA methylation and RNA transcriptome.

### Methyl-MiniSeq Genome-Wide bisulfite sequencing (GWBS)

Genomic DNA was isolated from the 17 fetal testicular specimens with the Quick-DNA/RNA Miniprep Plus Kit (Cat#: D7003, Zymo Research). The DNA samples were used for processing and analysis. Briefly, the Zymo Research DNA Clean & Concentrator™-5 (Cat#: D4003) was used to purify 500 ng of input genomic DNA after it had been successively digested with 60 units of Taqαl and 30 units of MspI (NEB). Following Illumina’s instructions, fragments were ligated to pre-annealed adapters that included 5ʹ-methyl-cytosine rather than cytosine. Using a 2.5% NuSieve 1:1 agarose gel, adaptor-ligated fragments ranging in size from 150–250 bp and 250–350 bp were extracted using the Zymoclean^TM^ Gel DNA Recovery Kit (Cat#: D4001). After that, the EZ DNA Methylation-Lightning^TM^ Kit (Cat#: D5030) was used to bisulfite-treat the fragments, and DNA Clean & Concentrator™-5 (Cat#: D4003) was used to purify the products of preparative-scale PCR. Methyl-MiniSeq libraries were sequenced using the Illumina, NovaSeq 6000 (San Diego, CA). TrimGalore 0.6.4 was then used to modify raw FASTQ files, fill in nucleotides, and trim quality. The impact of trimming and the overall quality distributions of the data were evaluated using FastQC 0.11.9. Trimmed reads were aligned to the Bos taurus ARS-UCD v1.2 reference genome (GCF_002263795.1) using Bismark 0.22.3. MethylDackel 0.5.0 was used to call the methylation and unmethylated read totals for every CpG site. Differentially methylated cytosines (DMCs) and regions (DMRs) were identified, annotated, and visualized using DSS through a comparative statistical analysis. Cytosines with read depth ≥ 5 in ≥ 2 samples per group were retained. DSS, which by default performed the Wald test and the Benjamini-Hochberg *P*-value adjustment, was used to identify DMCs and DMRs. FDR ≤ 0.05 and absolute methylation difference > 0.1 are considered significant DMCs and DMRs. Genes overlapping the DMRs were then subjected to a functional enrichment analysis using the g: Profiler tool. The DMCs and DMRs were also annotated with the closest expressed gene in the transcriptome dataset using bedtools v2.31.1 (https://doi.org/10.1093/bioinformatics/btq033) (Quinlan and Hall, 2010).

### Whole transcriptome analysis of fetal testicular tissue

The quick-RNA Mininprep Plus Kit (Cat#: R1057; Zymo Research) was used for isolation and purification of total RNA from the 17 fetal testicular tissue specimens. Libraries were constructed utilizing the Zymo-Seq RiboFree Total RNA Library Prep Kit (Cat # R3000) in accordance with the manufacturer’s instruction (v1.3.0). Briefly, RNA underwent reverse transcription to form cDNA, subsequently followed by the depletion of ribosomal RNA. Subsequently, the partial P7 adapter sequence was ligated to the 3ʹ end of cDNAs, followed by second strand synthesis and ligation of the partial P5 adapter to the 5ʹ end of the double-stranded DNAs. Finally, libraries were enhanced to include full-length adapters. The successful construction of the library was validated using Agilent’s D1000 ScreenTape Assay on the TapeStation. RNA-Seq libraries were sequenced on an Illumina platform to a minimum depth of 30 million read pairs per sample. The RNA-seq data were analyzed using the Zymo Research RNA-Seq pipeline, originally adapted from nf-core/rnaseq pipeline v1.4.2 (https://github.com/nf-core/rnaseq) (Patel et al., 2020) using Nextflow (https://www.nextflow.io/) (Di ­Tommaso et al., 2017). Briefly, quality control of raw reads was carried out using FastQC v0.11.9 (http://www.bioinformatics.babraham.ac.uk/projects/fastqc). Adapter and low-quality sequences were trimmed from raw reads using Trim Galore! v0.6.6 (https://www.bioinformatics.babraham.ac.uk/projects/trim_galore). Trimmed reads were aligned to the Bos taurus ARS-UCD v1.2 reference genome (Ensemble release 110, https://doi.org/10.1093/nar/gkad1049) (Peter et al., 2024) using STAR v2.6.1d (https://github.com/alexdobin/STAR) (Dobin et al., 2013). BAM file fconceptiltering and indexing was carried out using SAMtools v1.9 (https://github.com/samtools/samtools) (Danecek et al., 2021). RNAseq library quality control was implemented using RSeQC v4.0.0 (http://rseqc.sourceforge.net/) (Wang et al., 2012) and QualiMap v2.2.2-dev (http://qualimap.conesalab.org/) (García-Alcalde et al., 2012). Duplicate reads were marked using Picard tools v2.23.9 (http://broadinstitute.github.io/picard/) (Picard Toolkit, 2019). Library complexity was estimated using Preseq v2.0.3 (https://github.com/smithlabcode/preseq) (Daley and Smith, 2013). Duplication rate quality control was performed using dupRadar v1.18.0 (https://bioconductor.org/packages/dupRadar/) (Sayols et al., 2016). Reads overlapping with exons were assigned to genes using featureCounts v2.0.1 (http://bioinf.wehi.edu.au/featureCounts/) (Liao et al., 2014). Differential gene expression analysis was completed using DESeq2 v1.28.0 (https://bioconductor.org/packages/DESeq2/) (Love et al., 2014). Functional enrichment analysis was achieved using g: Profiler python API v1.0.0 (https://biit.cs.ut.ee/gprofiler/gost) (Raudvere et al., 2019). Quality control and analysis results plots were visualized using MultiQC v1.9 (https://github.com/ewels/MultiQC) (Ewels et al., 2016).

### Overlapping analysis of DNA methylation and gene expression

We investigated the relationship between DNA methylation and differential gene expression to evaluate the genomic effects of maternal dietary restriction, both with and without melatonin administration. The common genes between DMRs and DEGs across different treatment groups were identified by an overlap analysis using bedtools v2.31.1 (https://doi.org/10.1093/bioinformatics/btq033). The diagrams were made by the Venny 2.1 Online tool (Venny, 2007).

## Results

### Methyl-MiniSeq Genome-wide bisulfite sequencing (GWBS) of fetal testicular tissue

**Figure 1.**
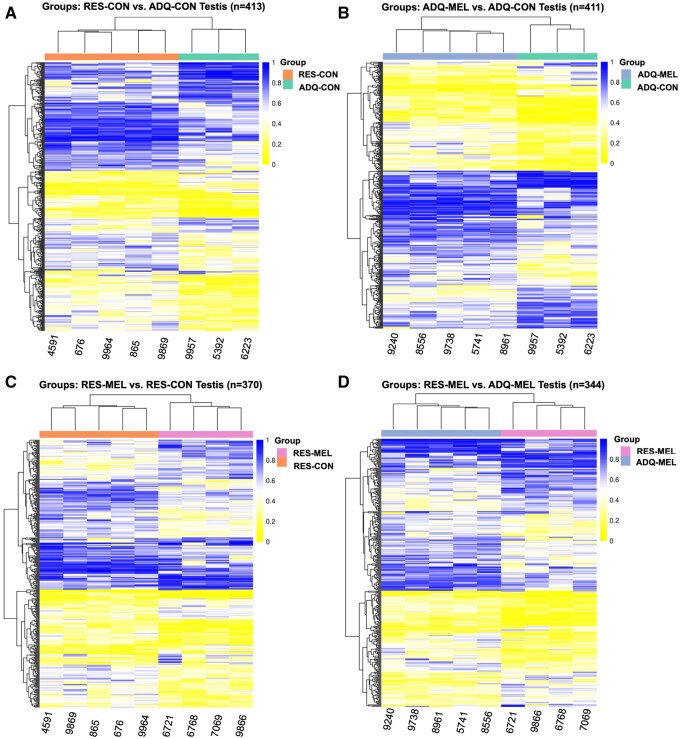
Heatmap showing the hierarchical clustering of differential methylation in the genomic regions of fetal testicular tissue among different treatment groups. (A) Differentially methylated regions (DMRs; *n *= 413) between RES-CON (orange) and ADQ-CON (green), (B) ADQ-MEL (grey) and ADQ-CON (green; *n* = 411), (C) RES-CON (orange) and RES-MEL (pink; *n* = 370), and (D) ADQ-MEL (gray) and RES-MEL (pink; *n* = 344). Significant DMRs have *FDR* ≤ 0.05 and *absolute methylation difference* ≥ 0.1. The row represents the gene, and the column denotes the sample. Yellow: hypomethylated genes; blue: hypermethylated genes. The darkness of each color corresponds to the magnitude of the differences vs. the mean value.

Sequencing of the bisulfite libraries from 17 fetal testicular tissue samples, resulted in an average CpG coverage of 13.26 million reads (ranging from 9.11 to 19.284 million reads). After trimming and mapping, 2,768,233 total methylated sites were found. The distribution of methylation values for all samples is illustrated in Supplementary Figure S1A, and the distribution of mean methylation values for each treatment group are illustrated in Supplementary Figure S1B. The four contrasts (RES-MEL vs. RES-CON, ADQ-MEL vs. ADQ-CON, RES-CON vs. ADQ-CON, and RES-MEL vs. ADQ-MEL) were used to examine DMCs, resulting in 4,891, 5,558, 5,418, and 4,407 significant DMCs (*FDR* < 0.05), respectively (Table 1). DMCs that are near each other are grouped into differentially methylated regions (DMRs), resulting in 413 significant DMRs for RES-CON vs. ADQ-CON, the highest DMRs number in between the treated groups (Figure 1A); 411 for ADQ-MEL vs. ADQ-CON (Figure 1B); 370 significant DMRs for RES-MEL vs. RES-CON (Figure 1C); and 344 for RES-MEL vs. ADQ-MEL (Figure 1D).

**Table 1.** Number of DMCs (differentially methylated CpGs sites) and DMRs (differentially methylated regions)

Type	Group	Input	Output	*P*adj ≤ 0.05	*P*adj ≤ 0.05 & methDiff ≤ −0.1	*P*adj ≤ 0.05 & methDiff ≥ 0.1
**DMC**	RES-MEL vs. RES-CON	1,696,648	5,062	4,891	2,942	1,949
**DMR**	RES-MEL vs. RES-CON	1,696,648	370	NA	225	145
**DMC**	ADQ-MEL vs. ADQ-CON	1,571,549	5,824	5,558	2,010	3,548
**DMR**	ADQ-MEL vs. ADQ-CON	1,571,549	411	NA	142	269
**DMC**	RES-CON vs. ADQ-CON	1,600,570	5,714	5,418	1,756	3,662
**DMR**	RES-CON vs. ADQ-CON	1,600,570	413	NA	127	285
**DMC**	RES-MEL vs. ADQ-MEL	1,666,164	4,590	4,407	2,656	1,751
**DMR**	RES-MEL vs. ADQ-MEL	1,666,164	344	NA	210	133

DMCs that are near each other are grouped into DMRs.

To further evaluate the genomic effects of nutritional restriction with and without melatonin supplementation on fetal testis development, we investigated the relationship between DNA methylation, gene annotations, and gene expression profiles. Differentially methylated genes (DMGs) were found by overlapping DMRs with annotated genomic regions, including genes, exons, introns, promoters, and CpG islands. The majority of the DMRs are located within the introns followed by CpG islands, exons, and promoters of annotated genes as shown in Table 2.

**Table 2.** Locations of annotated genomic regions on the differentially methylated overlapped genes

Comparisons	Promoters	Exons	Introns	GpC islands
**RES-Mel vs. ADQ-MEL**	25	89	183	83
**RES-CON vs. ADQ-CON**	18	94	219	117
**ADQ-MEL vs. ADQ-CON**	32	97	219	114
**RES-MEL vs. RES-CON**	24	99	189	116

### Functional enrichment of DMGs

DMRs in the promoter region have the capacity to affect gene transcription, whereas DMRs in the gene body region frequently exhibit a positive correlation with gene expression levels. This analysis of these DMGs can offer valuable insights into the regulatory functions of DNA methylation. Comparing RES-CON vs. ADQ-CON testicular tissues, there were 417 DMGs (Supplementary Table S1). Many of the top significant Gene Ontology (GO) terms appear to be involved in fetal testicular development as a main effect of nutrient restriction during mid and late gestation (examples included: anatomical structure development, multicellular organism development, developmental process, cell differentiation, system development and regulation of cell morphogenesis alongside with catalytic activities, as shown in Table 3). When comparing ADQ-MEL with ADQ-CON 420 DMGs were identified, some of which were hypomethylated (such as: Scavenger receptor class F member 1 [*SCARF1*], non-specific serine/threonine protein kinase [*TRIO*], and RNA binding protein fox-1 homolog 3 [*RBFOX3*] and others were hypermethylated (such as: Dachsous cadherin-related 2 [*DCHS2*], Collectin-11 [*COLEC11*], Tubulin tyrosine ligase like 8 [*TTLL8*], and Palladin [*PALLD*]) (Supplementary Table S2). These previously listed genes were involved mainly in animal organ development, positive regulation of biological process as reproductive process. These results indicated that nutritional alongside with melatonin supplementation are important for animal organs development. The contrast RES-MEL vs. RES-CON contained 377 DMGs. These DMGs were significantly enriched in GO terms such as phosphorus metabolic process, developmental process, and response to stimulus (Supplementary Table S3). Comparing RES-MEL with ADQ-MEL results in 348 DMGs, significantly enriched in positive regulation of biological process, regulation of developmental process, and regulation of response to stimulus (Table 3). In comparison between RES-MEL and ADQ-MEL, out of 345 differentially methylated genes, 196 exhibited overlaps (Supplementary Table S4). The majority of these genes participate in the regulation of biological and developmental processes, as well as responses to stimuli as designed in our study for maternal nutrition restriction during mid-late gestation (Table 3).

**Table 3.** The top significant GO terms of differently methylated gene (DMGs) sets among different treated groups

Treatment	GO: term name	Query *P*-value	Term size	GO: Term ID	Source
**RES-CON vs. ADQ-CON**	Regulation of signaling	1.57E−06	2591	GO: 0023051	GO: Biological Process
	Regulation of cell communication	4.03E−06	2587	GO: 0010646	GO: Biological Process
	Biological regulation	9.69E−05	10961	GO: 0065007	GO: Biological Process
	Anatomical structure development	1.04E−04	4390	GO: 0048856	GO: Biological Process
	Multicellular organism development	1.67E−04	3409	GO: 0007275	GO: Biological Process
	Developmental process	3.31E−04	4730	GO: 0032502	GO: Biological Process
	Cell–cell signaling	3.40E−04	1149	GO: 0007267	GO: Biological Process
	Cell adhesion	6.90E−04	1073	GO: 0007155	GO: Biological Process
	Regulation of biological process	8.22E−04	10636	GO: 0050789	GO: Biological Process
	Regulation of signal transduction	1.01E−03	2252	GO: 0009966	GO: Biological Process
	Positive regulation of biological process	1.60E−03	4972	GO: 0048518	GO: Biological Process
	Regulation of cellular process	1.70E−03	10155	GO: 0050794	GO: Biological Process
	Positive regulation of cellular process	3.96E−03	4528	GO: 0048522	GO: Biological Process
	Signaling	4.69E−03	5732	GO: 0023052	GO: Biological Process
	Cell communication	4.73E−03	5814	GO: 0007154	GO: Biological Process
	Cell junction organization	8.49E−03	556	GO: 0034330	GO: Biological Process
	Cell differentiation	1.03E−02	3138	GO: 0030154	GO: Biological Process
	Cellular developmental process	1.03E−02	3138	GO: 0048869	GO: Biological Process
	RHOA GTPase cycle	1.68E−02	128	REAC: R-BTA-8980692	Reactome
	Regulation of response to stimulus	2.46E−02	3007	GO: 0048583	GO: Biological Process
	System development	2.53E−02	2867	GO: 0048731	GO: Biological Process
	Cell junction assembly	2.96E−02	321	GO: 0034329	GO: Biological Process
	Nervous system development	3.14E−02	1727	GO: 0007399	GO: Biological Process
	Negative regulation of signal transduction	3.41E−02	995	GO: 0009968	GO: Biological Process
	Regulation of cell morphogenesis	4.91E−02	174	GO: 0022604	GO: Biological Process
**RES-Mel vs. ADQ-MEL**	Positive regulation of biological process	5.76E−03	4972	GO: 0048518	GO: Biological Process
	Regulation of biological process	6.02E−03	10636	GO: 0050789	GO: Biological Process
	Biological regulation	1.04E−02	10961	GO: 0065007	GO: Biological Process
	Regulation of developmental process	1.19E−02	1826	GO: 0050793	GO: Biological Process
	Regulation of cell communication	1.78E−02	2587	GO: 0010646	GO: Biological Process
	Regulation of signaling	1.85E−02	2591	GO: 0023051	GO: Biological Process
	Regulation of response to stimulus	3.75E−02	3007	GO: 0048583	GO: Biological Process
	Endocytosis	4.82E−02	230	KEGG: 04144	KEGG Pathway
**ADQ-MEL vs. ADQ-CON**	Animal organ development	3.13E−03	2214	GO: 0048513	GO: Biological Process
	Positive regulation of biological process	1.22E−02	4972	GO: 0048518	GO: Biological Process
**RES-MEL vs. RES-CON**	Phosphorus metabolic process	2.72E−02	1930	GO: 0006793	GO: Biological Process
	Regulation of signaling	2.92E−02	2591	GO: 0023051	GO: Biological Process
	Phosphate-containing compound metabolic process	4.75E−02	1906	GO: 0006796	GO: Biological Process

### Whole transcriptome analysis of fetal testicular tissue

**Figure 2.**
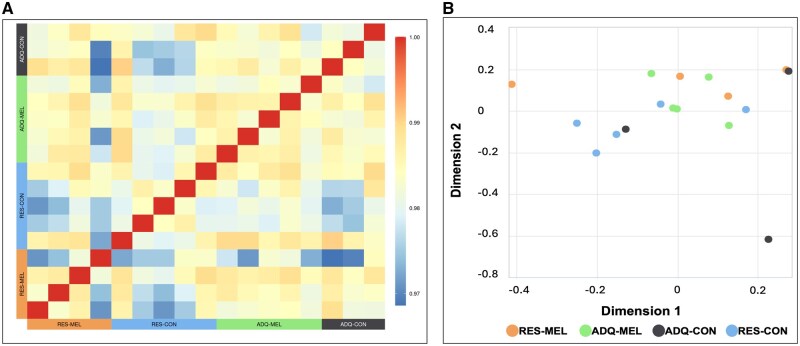
(A) Similarity matrix of fetal testicular tissue samples, the similarities were calculated using normalized and ‘rlog’ transformed read counts of all genes using DESeq2, minimum was 0.97 and maximum was 1.0. (B) Multidimensional scaling analysis of fetal testicular samples, adequately fed (ADQ-CON; 100% NRC recommendation, S9957, S6223, S5392), nutrient restricted (RES-CON, 60% NRC recommendation, S4591, S9964, S676, S9869, S865), and adequately fed or nutrient restricted supplemented with 20 mg/d of melatonin (ADQ-MEL, S8556, S5741, S9240, S9738, S8961; MEL-RES, S6721, S6768, S7059, S9866).

Transcriptome analysis was performed on 17 fetal testicular samples. The RNA-seq libraries were sequenced to an average depth of 46.17 million reads per sample (ranging from 38M in S5741 to 55.5M in S8556) with 48% average GC%. Trimming removed and average of 0.23% reads per sample. An average 99.77% (ranging from 99.70% to 99.80%) of the trimmed data mapped to the reference genome. After QC of these alignments, feature counts found 25,706 genes expressed. Correlations between each fetal testicular tissue sample are visualized in a heatmap (Figure 2A). Additionally, multidimensional scaling was conducted to visualize the distance/similarity between samples using the 500 genes with highest variance (Figure 2B).

**Figure 3.**
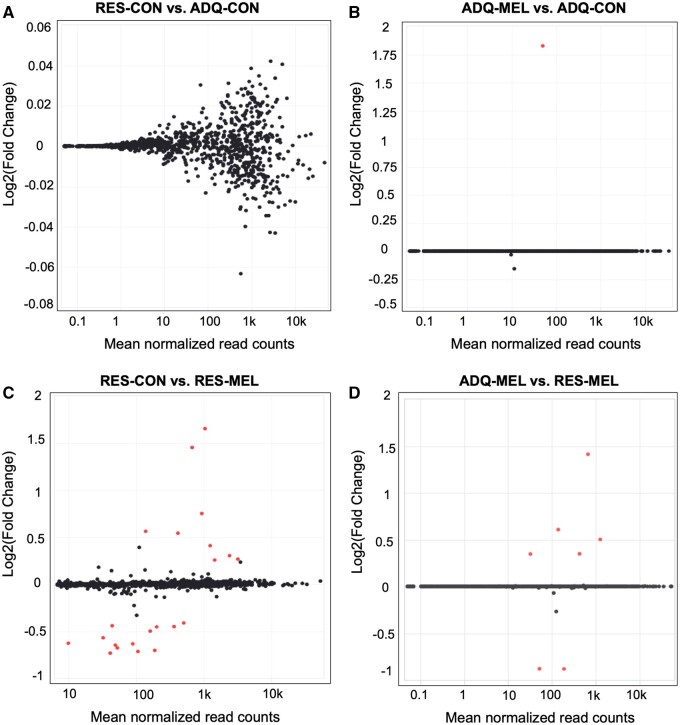
The MA plots for differentially expressed genes (up to the first 1,000 genes) and up to 1,000 randomly selected non-differentially expressed genes. Expression levels are shown on X-axis while Log2 of fold changes (Log2 FC) are shown on Y-axis. Red dots represent differentially expressed genes (adjusted *P-*values < 0.05) comparing between RES-CON and ADQ-CON testicular tissues (A), ADQ-MEL vs. ADQ-CON testicular tissue (B), RES-CON vs. RES-MEL (C), ADQ-MEL vs. RES-MEL (D). Grey dots represent non-differentially expressed genes.

DESeq2 was used to conduct statistical analysis of differential gene expression. Genes with Benjamini-Hochberg *adjusted P-values* (FDR) < 0.05 were considered differentially expressed. When comparing RES-CON with ADQ-CON (Supplementary Table S5), no significantly differentially expressed genes were found (Figure 3A). The ADQ-MEL vs. ADQ-CON comparison (Supplementary Table S6) had a single significantly (FDR = 0.0005) upregulated (log2 FC = 1.864) gene: Kynurenine—oxoglutarate transaminase 1 (*KYAT1*) (ENSBTAG00000036099; Figure 3B). In contrast, comparing RES-MEL and RES-CON (Supplementary Table S7) reveals 9 upregulated and 13 downregulated genes (Table 4; Figure 3C). Finally, five genes were upregulated and two downregulated in RES-MEL versus ADQ-MEL (Table 4; Figure 3D, Supplementary Table S8).

**Table 4.** Significant differentially expressed genes (DEGs) comparing among RES-MEL vs. RES-CON and RES-MEL vs. ADQ-MEL

Comparison	Gene ID	Gene abbreviation	log2FoldChange	Adjusted *P*-value
**RES-MEL vs. RES-CON**	ENSBTAG00000024420	COL28A1	1.9216	9.41E−07
	ENSBTAG00000022275	RPL10	1.7814	5.01E−05
	ENSBTAG00000050253	TRPM3	0.5793	0.0002
	ENSBTAG00000012848	PTPRU	−0.8186	0.0023
	ENSBTAG00000038168	SLITRK1	0.8908	0.0023
	ENSBTAG00000013401	ARHGEF40	0.3287	0.0053
	ENSBTAG00000034693	SYT1	0.4500	0.0122
	ENSBTAG00000049347	LOC112442676	−4.0405	0.0170
	ENSBTAG00000007698	TMEM59L	−0.9603	0.0196
	ENSBTAG00000005984	DAAM1	0.3202	0.0267
	ENSBTAG00000008274	ENSBTAG00000008274	−1.5941	0.0285
	ENSBTAG00000024086	TMEM35B	0.7129	0.0285
	ENSBTAG00000002999	ANAPC15	−0.5421	0.0285
	ENSBTAG00000003200	ENSBTAG00000003200	0.3188	0.0285
	ENSBTAG00000018064	FAM221A	−1.0836	0.0329
	ENSBTAG00000031238	SHCBP1L	−0.9366	0.0338
	ENSBTAG00000020352	PAQR5	−1.0338	0.0354
	ENSBTAG00000008511	PPP4R3C	−1.7128	0.0407
	ENSBTAG00000021072	DTNB	−0.5280	0.0450
	ENSBTAG00000051764	LncRNA	−1.9899	0.0463
	ENSBTAG00000003171	SHANK2	−0.7015	0.0489
	ENSBTAG00000004863	RIC3	−0.6131	0.0489
**RES-MEL vs. ADQ-MEL**	ENSBTAG00000012848	PTPRU	−0.9522	0.0001
	ENSBTAG00000022275	RPL10	1.7182	0.0002
	ENSBTAG00000034693	SYT1	0.5203	0.0011
	ENSBTAG00000011881	TDRD10	−1.3270	0.0290
	ENSBTAG00000024086	TMEM35B	0.7599	0.0338
	ENSBTAG00000052713	H2B	2.9672	0.0493
	ENSBTAG00000050253	TRPM3	0.4598	0.0493

### Overlapping analysis of DNA methylation and gene expression

**Figure 4.**
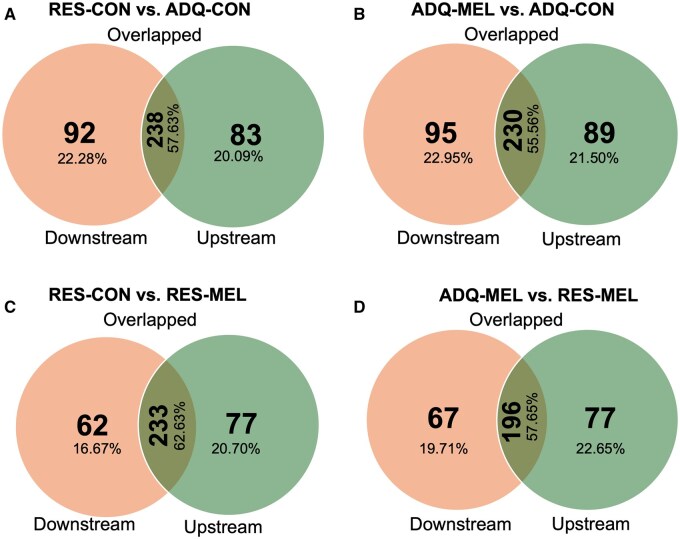
Venn diagrams for the numbers and locations of methylated regions of the expressed genes. The orange color indicated that the methylation region is downstream of the gene, while the green color indicated that the methylation region is upstream of the gene, the olive green means the expressed gene and methylation region overlap for each comparsion. (A) RES-CON vs. ADQ-CON; (B) ADQ-MEL vs. ADQ-CON; (C) RES-CON vs. RES-MEL; (D) ADQ-MEL vs. RES-MEL.

**Figure 5.**
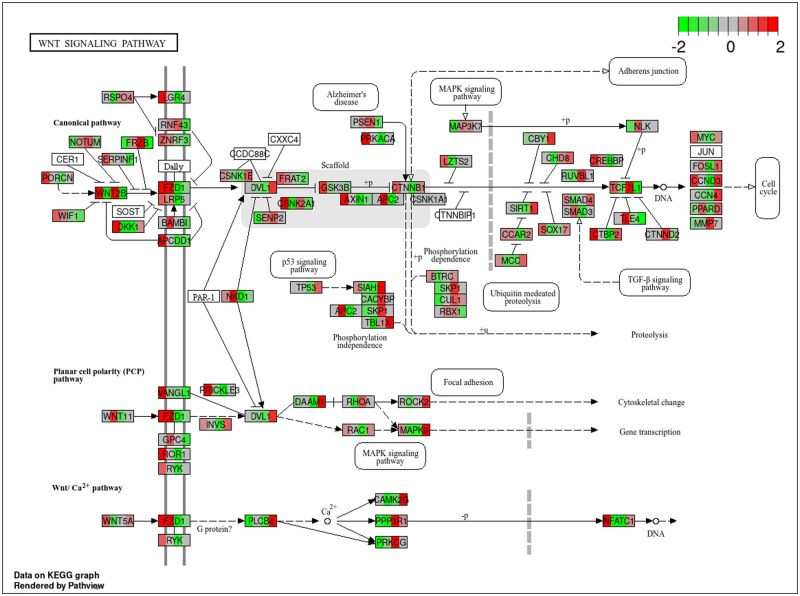
WNT signaling pathway with the expression data mapped to it. Pathway information generated by Kyoto Encyclopedia of Genes and Genomes (KEGG). The graph shows the gene-wise normalized expression (z score) for each group (ADQ-CON, ADQ-MEL, RES-CON, and RES-MEL). Because of the normalization, the expression data is comparable within a gene but not across genes.

To assess the genomic impacts of maternal nutritional restriction, both with and without melatonin supplementation, we examined the correlation between DNA methylation and gene expression. An overlap analysis was performed to determine the common genes between DMRs and DEGs across various treatment groups. We identified 238 (57.63%) overlapping genes between the RES-CON vs. ADQ-CON fetal testicular tissue, 230 (55.56%) overlapping genes in the ADQ-MEL vs. ADQ-CON fetal testicular tissue, the highest percentage (62.63%) of overlapped genes (233) were identified for RES-CON vs. RES-MEL fetal testicular tissue, and 196 genes in the ADQ-MEL vs. RES-MEL fetal testicular tissue (Figure 4A–D). The detailed list for overlapped genes for all treated groups is listed in Supplementary Tables S1–S4. Interestingly, Disheveled-associated activator of morphogenesis 1 (*DAAM1*), in RES-MEL vs. RES-CON, was the only overlapped statistically significant differentially expressed gene DEG (log2 FC = 0.320) that was also differentially methylated (methylation difference −0.2091) (Supplementary Table S3). Interestingly, this gene is involved in Wnt pathway as illustrated in Figure 5.

## Discussion

Although it is well known that maternal nutrition can affect fetal development in both males and females, most recent studies focus mainly on female offsprings. Consequently, male developmental programming remains inadequately comprehended. Numerous maladaptive characteristics may result from inadequate maternal nutrition in offspring. Therefore, this investigation postulated that the epigenomic and transcriptome patterns of fetal testicular tissue, and the developmental programming of male fetuses, would be altered by maternal nutrient restriction and melatonin supplementation.

In ruminants 60% to 90% of fetal growth occurs during the last third of gestation (Reynolds and Redmer, 1995). Male offspring exhibit elevated energy requirements for growth and may be more susceptible to maternal nutrition restriction (Hewison and Gaillard, 1999). Bull Sertoli cell counts rise from mid-gestation until birth (Hochereau-de Reviers et al., 1987) and continue to rise through puberty (Andrade et al., 2013), rendering maternal nutritional restriction during this window more detrimental than early gestational intervention. In this study we commenced our experiment around mid to late gestation, specifically on day 160, to align with testicular function and sexual differentiation, ending on day 240 of gestation. Melatonin diminishes testicular reactive oxygen species (ROS) and inflammation (Frungieri et al., 2017, El Shalofy et al., 2021). Melatonin upregulated spermatogenesis-related genes like Cyclin D1, Cyclin E, Dhh, Occludin, and Claudin in bovine Sertoli cells (Yang et al., 2014). Melatonin suppresses 8-OH-deoxyguanosine synthesis, activates p53, and enhances the expression of repair and anti-apoptotic proteins, thereby protecting DNA (Slominski et al., 2017; Skobowiat, 2018). Melatonin has the potential to boost the embryonic development progress through several mechanisms including DNA demethylation of pluripotency related gene (*Oct4*), DNA methylation maintenance of imprinted gene *H19/Igf2*, and the remethylation of tissue-specific gene *Thy1* in cloned embryos (Qu et al., 2020). It is well known that DNA hypermethylation in the promoter regions silencing gene expression. However, Namous et al. (2018) also found that DNA hypermethylation in the gene body influences expression either by silencing or enhancement. In this study most of the DMRs were found in the introns, then the exons, and lastly the promoters of DMGs. These distinct methylation alteration sites identified in this study influenced the significant expression patterns via either upregulation or downregulation. Comparing RES-CON with ADQ-CON, exhibited the highest DMRs frequency among the treated groups. It has been shown that a dam’s diet can alter the epigenetic status of her offspring during embryonic development.

An integrative analysis of the methylome and transcriptome profiles of fetal testicular tissues revealed a remarkable overlap between DMGs and DEGs. Interestingly, a comparison between RES-MEL and RES-CON indicated the key roles of melatonin supplementation in nutritionally restricted dams and compromised pregnancies. Nine genes (*DAAM1*, Collagen alpha-1(XXVIII) chain (*COL28A1*), Large ribosomal subunit protein uL16 (*RPL10*), Transient receptor potential cation channel subfamily M member 3 (*TRPM3*), SLIT and NTRK like family member 1 (*SLITRK1*), Rho guanine nucleotide exchange factor 40 (*ARHGEF40*), Synaptotagmin-1 (*SYT1*), Transmembrane protein 35B (*TMEM35B*), and chondroitin sulfate proteoglycan family member 4B (*CSPG4B*) demonstrate significantly elevated expression in the RES-MEL. Among these significant genes, only the *DAAM1* gene, located on chromosome 10, was hypomethylated. Other genes overlapped by DMRs include the hypomethylated genes Diacylglycerol kinase (*DGKD*), H2.0-like homeobox protein (*HLX*), Follistatin like 4 (*FSTL4*), histone H3-lysine (9) N-methyltransferase (*EHMT*)*1*, and TNF receptor superfamily member 19 (*TNFRSF19*), and hypermethylated genes Palmitoyltransferase (*ZDHHC8*) and Muellerian-inhibiting factor (*AMH*). Those overlapped genes were involved in phosphorus metabolic process, developmental processes, and responses to stimuli.

*DAAM1* is a member of the formin family which modulates the nucleation of linear actin filaments. The cytoskeleton comprising microfilaments, intermediate filaments, and microtubules, is indispensable to the survival of cells. The cytoskeleton is involved in a variety of processes, including intracellular trafficking, division, migration, motility, adhesion, differentiation, and cell shape determination (Moujaber and Stochaj, 2020). The dramatic cytoskeletal-dependent phenomenon may be directly influenced by *DAAM1*. Cytoskeleton remodeling is fundamental to spermatogenesis and, consequently, male fertility; thus, comprehending the various proteins and mechanisms that regulate their dynamics is critically important (Li et al., 2018). Thus, *DAAM1* regulates actin remodeling in the cytoplasm of germ cells throughout the differentiation phases of the seminiferous cycle and facilitates actin polymerization in type B spermatogonia nuclei, which is essential for DNA replication and cell division (Venditti et al., 2020). The Wnt signaling pathway is an evolutionarily conserved pathway that controls a variety of critical phases during embryonic development, including cell fate determination, cell polarity, cell migration, neural patterning, and organogenesis (Mezzacappa et al., 2012). It can be divided into two categories: canonical pathway and noncanonical pathway. *DAAM1* is activated and functions within noncanonical Wnt signaling also referred to as the planar cell polarity (PCP) pathway, which facilitates the activation of the small GTPase Rho to modulate gastrulation movements during embryogenesis and testis development (Habas et al., 2001; Park et al., 2006), as well as organogenesis (Li et al., 2011; Bao et al., 2012; Ajima et al., 2015). PCP modulates cell motility by modifying the actin cytoskeleton (Wallingford et al., 2002; Veeman et al., 2003). Pariante et al. (2016) investigated *DAAM1* during the post-natal development of the rat testis and in rat and human sperm, indicating its potential involvement in reproduction. Melatonin has been found to protects against Cadmium-induced testicular damage by regulating *DAAM1* expression (Venditti et al., 2021). Melatonin improves testicular health in rats; but further research is needed to prove its efficacy in bovine to produce high-quality spermatozoa for reproduction.

*COL28A1*, *RPL10*, *TRPM3*, *SLITRK1*, *ARHGEF40*, *SYT1*, *TMEM35B*, and *CSPG4B* exhibited significant elevated expression in the RES-MEL relative to RES-CON. The *COL28A1* gene encodes the collagen XXVIII protein, which is essential components of the testicular basal lamina, which plays a key role in regulating cell development and activity (Rossitto et al., 2022).

*RPL10* upregulation in RES-MEL compared with RES-CON regulates cell proliferation and development. *RPL10* expression levels were similar in spermatogonia and early spermatocytes, but significantly decreased in late spermatocytes and round spermatids, indicating transcriptional suppression during and after meiotic chromosomal inactivation. *RPL10l* compensates for *RPL10* silencing during spermatogenesis (Long et al., 2017). The variable expression of RP paralogues as *RPL10L* is crucial for ribosomal heterogeneity in spermatogenesis (Li et al., 2022). *TRPM3* and *SYT1* are important for the facilitated diffusion of a calcium (Ca^2+^) ions through a transmembrane aqueous pore or channel and the cellular response to Ca^2+^ ions, respectively. Ca^2+^ signaling is a significant modulator of cell activities, particularly involving the endoplasmic reticulum (ER) (Burgoyne et al., 2015). ER-mediated Ca^2+^ signaling may be of major significance during junction turnover associated with spermatocyte translocation and sperm release in the seminiferous epithelium (Vogl et al., 2018).

Conversely, 13 genes (Receptor-type tyrosine-protein phosphatase U (*PTPRU*), *snRNP-E*, Transmembrane protein 59-like (*TMEM59L*), Mucin 5B, oligomeric mucus/gel-forming (*MUC5B*), Anaphase-promoting complex subunit 15 (*ANAPC15*), family with sequence similarity 221 member A (*FAM221A*), Testicular spindle-associated protein SHCBP1L (*SHCBP1L*), Progestin and adipoQ receptor family member 5 (*PAQR5*), Protein phosphatase 4 regulatory subunit 3C (*PPP4R3C*), Dystrobrevin (*DTNB*), *long non-coding RNA* (*LncRNA*), SH3 and multiple ankyrin repeat domains 2 (*SHANK2*), RIC3 acetylcholine receptor chaperone (*RIC3*) exhibited greater expression in the RES-CON vs. RES-MEL without any differential methylation changes. Most of these genes exhibited some functions involved in embryonic developmental abnormalities and preterm birth linked to maternal undernutrition. Moreover, most of these genes are implicated in male fetal development and spermatogenesis. Protein phosphatase 4 (PP4) is a crucial in numerous essential cellular pathways, encompassing the DNA damage response which comprises DNA repair, cell cycle regulation, and apoptosis (Park and Lee, 2020). Gene ontology analysis for testicular *SHCBP1L* suggested its involvement in male meiosis cytokinesis, and spermatogenesis collaborating with *HSPA2* to sustain spindle integrity during meiosis in male germ cells. *SHCBP1L* is expressed in male germ cells in the testis, but not in female germ cells and mature sperm. Therefore, *SHCBP1L* is essential for germ cell division and spermatogenesis in mammals (Liu et al., 2014).

The significant DMGs of compromised pregnant dams in RES-CON relative to ADQ-CON have significant function in fetal testicular development (i.e., anatomical structure development, multicellular organism development, developmental process, cell differentiation, system development, regulation of cell morphogenesis, and catalytic activities). Interestingly, certain DMGs implicated in fetal testicular development exhibit hypomethylated (Neurogenic locus notch homolog protein 1 (*NOTCH1*), Rho GTPase-activating protein 7 (*DLC1*), Carbohydrate sulfotransferase (*CHST12*), A-kinase anchoring protein 13 (*AKAP13*), and TBC1 domain family member 22A (*TBC1D22A*), while others demonstrate hypermethylated (Rho guanine nucleotide exchange factor 19 (*ARHGEF19*), Insulin Like Growth Factor 2 Receptor (*IGF2R*), Transmembrane ascorbate-dependent reductase CYB561 (*CYB561*), Tumor necrosis factor ligand superfamily member 8 (*TNFSF*)*8*, and Peptidyl-prolyl cis-trans isomerase (*PPIF*). This aligns with the findings of Paradis et al. (2017), which indicate the potential consequences of insufficient maternal nutrition and the possible regulators of modified gene expression patterns, such as DNA methylation of imprinted genes like Insulin Like Growth Factor 2 (IGF2) and IGF2R, as well as microRNA expression. The murine models offer a new tool for identifying essential variables for gonocyte maintenance in the male embryonic gonad post sex determination and indicate a potential functional role for NOTCH signaling exclusively in Sertoli cells. Additionally, NOTCH signaling controls the maintenance and differentiation of Leydig progenitor cells during embryonic development (Tang et al., 2008). Research has revealed that AKAPs are crucial in regulating metabolism, growth, development, and reproduction (Ng et al., 2019; Melick et al., 2020).

The expression of *KYAT1* gene was significantly increased in the ADQ-MEL relative to the ADQ-CON group without alterations in methylation for this gene. The kynurenine pathway is implicated in various activities, such as antioxidative mechanisms (Xu et al., 2018), modulation of apoptosis and endothelial dysfunction (Wang et al., 2014). These mechanisms are essential factors in placental development and vital for a successful pregnancy. Furthermore, a comparison of ADQ-MEL with ADQ-CON revealed certain genes that were hypomethylated (*SCARF1*, *TRIO*, and *RBFOX3*) and others that were hypermethylated (*DCHS2*, *COLEC11*, Retinoic acid receptor beta (*RARB*), *TTLL8*, and *PALLD*). These genes were not significantly expressed, yet they were primarily engaged in animal organ development, and the positive regulation of biological process as reproduction. Crucially, depending on the underlying genetic sequence, DNA methylation in various genomic locations may have varying effects on gene activity (Moore et al., 2013).

Lastly, five genes exhibited significant greater expression in RES-MEL relative to ADQ-MEL, including Histone type 2-B (*H2B*) located on chromosome 23. Additionally, two genes (*PTPRU*, Tudor domain containing 10 (*TDRD10*) exhibited significant elevated expression in ADQ-MEL vs. RES-MEL without showing any differential methylation alternations for those genes. Some hypomethylated genes (E3 ubiquitin-protein ligase LNX (*LNX1*), and Complement component C6 (*C6*), and hypermethylated genes (Paralemmin-1 (*PALM*), Transporter (*SLC6A3*), Pleckstrin homology and RhoGEF domain containing G5 (*PLEKHG5*), and Phosphoribosyl pyrophosphate synthase-associated protein 1 (*PRPSAP1*)) are involved in positive regulation of biological processes, regulation of developmental process, and regulation of responses to stimuli. In rats, the meiotic phase of spermatogenic differentiation involves the expression of the testis-specific H2B (*TH2B*) histone gene. There exists a substantial correlation between the expression of the *TH2B* gene in germ cells and DNA hypomethylation (Choi and Chae, 1991). In spermatids, protamines replace nucleosomal histones to form a more compact chromatin architecture, which is essential for enhancing sperm motility and protecting DNA during sperm maturation (Oliva, 2006; Qian et al., 2013). Besides its biological role in sperm development, differentiation, motility, nucleosome assembly, chromatin remodeling, and spermatid nucleus differentiation, *TH2B* is significantly correlated with gene ontology in relation to molecular processes in chromatin and DNA binding (Kutchy et al., 2017). The distribution of *TH2B* was varied throughout all chromosomes, excluding the sex chromosomes (Patankar et al., 2021). *PTPs* dephosphorylate phosphate groups from protein tyrosine residues to modulate many cellular signaling pathways via their catalytic activity. The deletion or overexpression of several PTPs adversely affects normal pregnancy and embryonic development, resulting in reproductive disorders and compromised early embryonic development (Du et al., 2023). The piRNA pathway and gametogenesis are closely linked to the majority of the 12 mammalian TDRD proteins among them *TDRD10*, which have either male germline-specific or germline-enriched expression patterns (Jin et al., 2009; Siomi et al., 2010). DNA methylation has been linked to gene regulation and cell differentiation, according to multiple studies (Holliday and Pugh, 1975; Compere and Palmiter, 1981).

Many of these DMGs and DEGs gene sets altered in response to melatonin supplementation are implicated in biological and developmental processes and pathways. It is now well acknowledged that one of the main epigenetic factors affecting gene activity is DNA methylation, working in tandem with other regulators. These results suggested that melatonin, a cost-effective supplement, is crucial for fetal development and beneficial regulation of biological process for compromised pregnancy in cattle. However, there exists a limitation in this study, especially within the ADQ-CON group, *n* = 3, hence elevating the probability of a false positive for a gene. Moreover, future research should focus on the long-term effects of these epigenetic changes on bull development and reproductive efficiency. These new findings on developmental programming’s maternal effects emphasize the need to prioritize male prenatal development.

## Conclusion

This study indicates that melatonin supplementation during mid- to late-gestation positively affects the fetal testicular tissue epigenome and transcriptome. These findings suggest that male reproductive potential can be programmed, but adult testicular morphology and semen quality studies are needed. The integration of DMGs and DEGs in the omics profiles of fetal testicular investigation showed little overlap in significant expression at the time of sampling. Nonetheless, the overlapping genes identified in the DMGs and DEGs, such as the DAAM1 gene, offer a valuable opportunity for future research to investigate their relevance in the growth and maturation of the testes in male calves from birth to puberty. Epigenetics provide a new perspective on embryonic development and understanding how melatonin and/or dietary restriction affect epigenetic modifications is crucial.

## Supplementary Material

skaf455_Supplementary_Data

## Abbreviations:

ADQ-CON: adequately fed
RES-CON: nutrient restricted
ADQ-MEL: adequately fed supplemented with melatonin
RES-MEL: nutrient restricted ­supplemented with melatonin
DMR: differentially methylated region
DMG: differentially methylated gene

## References

[skaf455-B1] Ajima R. , Bisson J. A., Helt J. C., Nakaya M. A., Habas R., Tessarollo L., He X., Morrisey E. E., Yamaguchi T. P., Cohen E. D. 2015. DAAM1 and DAAM2 are co-required for myocardial maturation and sarcomere assembly. Dev. Biol. 408(1):126–139. 10.1016/j.ydbio.2015.10.003 26526197 PMC4765503

[skaf455-B2] Andrade L. P. , Rhind S. M., Rae M. T., Kyle C. E., Jowett J., Lea R. G. 2013. Maternal undernutrition does not alter sertoli cell numbers or the expression of key developmental markers in the mid-gestation ovine fetal testis. J. Negat. Results Biomed. 12:2. 10.1186/1477-5751-12-2 23295129 PMC3584724

[skaf455-B3] Bao B. , Zhang L., Hu H., Yin S., Liang Z. 2012. Deletion of a single-copy DAAM1 gene in congenital heart defect: a case report. BMC Med. Genet. 13:63. 10.1186/1471-2350-13-63 22857009 PMC3482563

[skaf455-B4] Brockus K. E. , Hart C. G., Gilfeather C. L., Fleming B. O., Lemley C. O. 2016. Dietary melatonin alters uterine artery hemodynamics in pregnant holstein heifers. Domest. Anim. Endocrinol. 55:1–10. 10.1016/j.domaniend.2015.10.006 26641925

[skaf455-B5] Burgoyne T. , Patel S., Eden E. R. 2015. Calcium signaling at ER membrane contact sites. Biochim. Biophys. Acta. 1853(9):2012–2017. 10.1016/j.bbamcr.2015.01.022. 25662816

[skaf455-B6] Caton J. S. , Crouse M. S., Reynolds L. P., Neville T. L., Dahlen C. R., Ward A. K., Swanson K. C. 2019. Maternal nutrition and ­programming of offspring energy requirements. Transl Anim Sci. 3(3):976–990. 10.1093/tas/txy127. 32704862 PMC7200455

[skaf455-B7] Choi S. W. , Friso S. 2010. Epigenetics: a new bridge between nutrition and health. Adv. Nutr. 1(1):8–16. 10.3945/an.110.1004 22043447 PMC3042783

[skaf455-B8] Choi Y. C. , Chae C. B. 1991. DNA hypomethylation and germ cell-specific expression of testis-specific H2B histone gene. J. Biol. Chem. 266(30):20504–20511. 10.1016/S0021-9258(18)54953-X. 1718964

[skaf455-B9] Compere S. J. , Palmiter R. D. 1981. DNA methylation controls the inducibility of the mouse metallothionein-I gene lymphoid cells. Cell. 25(1):233–240. 10.1016/0092-8674(81)90248-8 6168387

[skaf455-B10] Contreras-Correa Z. E. , Messman R. D., Sanchez-Rodriguez H., Burnett D. D., Lemley C. O. 2022. 395 Young scholar award talk: Melatonin: a promising therapeutic for compromised pregnancies. J Anim Sci. 100(Suppl_3):201–202. 10.1093/jas/skac247.366

[skaf455-B11] Contreras-Correa Z. E. , Messman R. D., Sidelinger D. R., King E. H., Sanchez-Rodriguez H. L., Burnett D. D., Lemley C. O. 2021. Melatonin alters bovine uterine artery hemodynamics, vaginal temperatures and fetal morphometrics during late gestational nutrient restriction in a season-dependent manner. J. Anim. Sci. 99(9):1–14. 10.1093/jas/skab242 PMC8420683 34387666

[skaf455-B12] Costa T. C. , Du M., Nascimento K. B., Galvão M. C., Meneses J. A. M., Schultz E. B., Gionbelli M. P., Duarte M. D. S. 2021. Skeletal muscle development in postnatal beef cattle resulting from maternal protein restriction during Mid-Gestation. Animals. (Basel). 11(3):860. 10.3390/ani11030860. 33803518 PMC8003034

[skaf455-B13] Daley T. , Smith A. D. 2013. Predicting the molecular complexity of sequencing libraries. Nat. Methods. 10(4):325–327. 10.1038/nmeth.2375 23435259 PMC3612374

[skaf455-B14] Danecek P. , Bonfield J. K., Liddle J., Marshall J., Ohan V., Pollard M. O., Whitwham A., Keane T., McCarthy S. A., Davies R. M. et al. 2021. Twelve years of SAMtools and BCFtools. Gigascience. 10(2). 10.1093/gigascience/giab008 PMC7931819 33590861

[skaf455-B15] Di Tommaso P. , Chatzou M., Floden E. W., Barja P. P., Palumbo E., Notredame C. 2017. Next flow enables reproducible computational workflows. Nat. Biotechnol. 35(4):316–319. 10.1038/nbt.3820 28398311

[skaf455-B16] Dobin A. , Davis C. A., Schlesinger F., Drenkow J., Zaleski C., Jha S., Batut P., Chaisson M., Gingeras T. R. 2013. STAR: ultrafast universal RNA-seq aligner. Bioinformatics. 29(1):15–21. 10.1093/bioinformatics/bts635 23104886 PMC3530905

[skaf455-B17] Du R. H. , Chen H. Y., Gao L. 2023. Roles of protein tyrosine phosphatases in reproduction and related diseases. Reprod. Dev. Med. 7(4):252–256. 10.1097/RD9.0000000000000064

[skaf455-B18] El Shalofy A. , Hedia M., Kastelic J. 2021. Melatonin improves testicular hemodynamics, echotexture and testosterone production in ossimi rams during the breeding season. Reprod. Domest. Anim. 56:1456–1463. 10.1111/rda.14010 34459033

[skaf455-B19] Ewels P. , Magnusson M., Lundin S., Käller M. 2016. MultiQC: summarize analysis results for multiple tools and samples in a single report. Bioinformatics. 32(19):3047–3048. 10.1093/bioinformatics/btw354 27312411 PMC5039924

[skaf455-B20] Frungieri M. B. , Calandra R. S., Rossi S. P. 2017. Local actions of melatonin in somatic cells of the testis. Int. J. Mol. Sci. 18(6):1170. 10.3390/ijms18061170. 28561756 PMC5485994

[skaf455-B21] García-Alcalde F. , Okonechnikov K., Carbonell J., Cruz L. M., Götz S., Tarazona S., Dopazo J., Meyer T. F., Conesa A. 2012. Qualimap: evaluating next- generation sequencing alignment data. Bioinformatics. 28(20):2678–2679. 10.1093/bioinformatics/bts503 22914218

[skaf455-B22] Godfrey K. M. , Barker D. J. 2001. Fetal programming and adult health. Public Health Nutr. 4(2B):611–624. 10.1079/phn2001145 11683554

[skaf455-B23] González-Arto M. , Aguilar D., Torrubia E. G., Gallego M., Carvajal-Serna M., Herrera-Marcos L. V., Serrano-Blesa E., Santos Hamilton D, Pérez-Pé T. R., Muiño-Blanco R. et al. 2017. Melatonin MT1 and MT2receptors in the ram reproductive tract. Int. J. Mol. Sci. 18(3):662. 10.3390/ijms18030662. 28335493 PMC5372674

[skaf455-B24] Habas R. , Kato Y., He X. 2001. Wnt/frizzled activation of rho regulates vertebrate gastrulation and requires a novel formin homology protein Daam1. Cell. 107(7):843–854. 10.1016/s0092-8674(01)00614-6 11779461

[skaf455-B25] Hewison A. J. , Gaillard J. M. 1999. Successful sons or advantaged daughters? The Trivers-Willard model and sex-biased maternal investment in ungulates. Trends Ecol. Evol. 14(6):229–234. 10.1016/s0169-5347(99)01592-x 10354625

[skaf455-B26] Hochereau-de Reviers M. T. , Monet-Kuntz C., Courot M. 1987. Spermatogenesis and sertoli cell numbers and function in rams and bulls. J. Reprod. Fertil. Suppl. 34:101–14. https://api.semanticscholar.org/CorpusID:35105512 3305912

[skaf455-B27] Holliday R , Pugh J. E. 1975. DNA modification mechanisms and gene activity during development. Science. 187(4173):226–232. 10.1126/science.187.4173.226 1111098

[skaf455-B28] Jaenisch R. , Bird A. 2003. Epigenetic regulation of gene expression: how the genome integrates intrinsic and environmental signals. Nat. Genet. 33 Suppl:245–254. 10.1038/ng1089 12610534

[skaf455-B29] Ji Y. , Wu Z., Dai Z., Sun K., Wang J., Wu G. 2016. Nutritional epigenetics with a focus on amino acids: implications for the development and treatment of metabolic syndrome. J. Nutr. Biochem. 27:1–8. 10.1016/j.jnutbio.2015.08.003 26427799

[skaf455-B30] Jin J. , Xie X., Chen C., Park J. G., Stark C., James D. A., Olhovsky M., Linding R., Mao Y., Pawson T. 2009. Eukaryotic protein domains as functional units of cellular evolution. Sci. Signal. 2(98):ra76. 10.1126/scisignal.2000546 19934434

[skaf455-B31] Korkmaz A , Reiter R. J. 2008. Epigenetic regulation: a new research area for melatonin? J. Pineal Res. 44(1):41–44. 18078446 10.1111/j.1600-079X.2007.00509.x

[skaf455-B32] Kutchy N. A. , Velho A., Menezes E. S. B., Jacobsen M., Thibaudeau G., Wills R. W., Moura A., Kaya A., Perkins A., Memili E. 2017. Testis specific histone 2B is associated with sperm chromatin dynamics and bull fertility-a pilot study. Reprod. Biol. Endocrinol. 15(1):59. 10.1186/s12958-017-0274-1. 28764714 PMC5539985

[skaf455-B33] Lan X. , Cretney E. C., Kropp J., Khateeb K., Berg M., Peñagaricano F., Magness R., Radunz A., Khatib H. 2013. Maternal diet during pregnancy induces gene expression and DNA methylation changes in fetal tissues in sheep. Front. Genet. 4:49. 10.3389/fgene.2013.00049. 23577020 PMC3617393

[skaf455-B34] Lemley C. O. 2020. Fetal programming: maternal-fetal interactions and postnatal performance. Clin. Theriogenology. 12(3):252–267. https://clinicaltheriogenology.net/index.php/CT/article/view/9240.

[skaf455-B35] Lemley C. O. , Camacho L. E., Vonnahme K. A. 2013. Uterine in- fusion of melatonin or melatonin receptor antagonist alters ovine feto-placental hemodynamics during midgestation. Biol. Reprod. 89(2):40. 10.1095/biolreprod.113.109074. 23782836

[skaf455-B36] Lemley C. O. , Meyer A. M., Camacho L. E., Neville T. L., Newman D. J., Caton J. S., Vonnahme K. A. 2012. Melatonin supplementation alters uteroplacental hemodynamics and fetal development in an ovine model of intrauterine growth restriction. Am. J. Physiol. Regul. Integr. Comp. Physiol. 302(4):R454–R467. 10.1152/ajpregu.00407.2011. 22129617

[skaf455-B37] Li D. , Hallett M. A., Zhu W., Rubart M., Liu Y., Yang Z., Chen H., Haneline L. S., Chan R. J., Schwartz R. J. et al. 2011. Dishevelled-associated activator of morphogenesis 1 (Daam1) is required for heart morphogenesis. Development. 138(2):303–315. 10.1242/dev.055566 21177343 PMC3005605

[skaf455-B38] Li H. , Huo Y., He X., Yao L., Zhang H., Cui Y., Xiao H., Xie W., Zhang D., Wang Y. et al. 2022. A male germ-cell-specific ribosome controls male fertility. Nature. 612(7941):725–731. 10.1038/s41586-022-05508-0 36517592

[skaf455-B39] Li L. , Mao B., Wu S., Lian Q., Ge R. S., Silvestrini B., Cheng C. Y. 2018. Regulation of spermatid polarity by the actin- and microtubule (MT)-based cytoskeletons. Semin. Cell Dev. Biol. 81:88–96. 10.1016/j.semcdb.2018.01.013 29410206 PMC6078802

[skaf455-B40] Liao Y. , Smyth G. K., Shi W. 2014. Feature counts: an efficient general-purpose program for assigning sequence reads to genomic features. Bioinformatics. 30(7):923–930. 10.1093/bioinformatics/btt656 24227677

[skaf455-B41] Liu M. , Shi X., Bi Y., Qi L., Guo X., Wang L., Zhou Z., Sha J. 2014. Jun; SHCBP1L, a conserved protein in mammals, is predominantly expressed in male germ cells and maintains spindle stability during meiosis in testis. Mol. Hum. Reprod. 20(6):463–475. 10.1093/molehr/gau014. 24557841

[skaf455-B42] Long J. , Tao L., Xingxia Z., Beibei Z., Changping Y., Yang L., Suixing F., Xiaohua J., Teka K., Qiaomei H. et al. 2017. RPL10L is required for male meiotic division by compensating for RPL10 during meiotic sex chromosome inactivation in mice. Current Biology. 27(10):1498–1505.e6. 10.1016/j.cub.2017.04.017. 28502657

[skaf455-B43] Long J. M. , Trubenbach L. A., Hobbs K. C., Poletti A. E., Steinhauser C. B., Pryor J. H., Long C. R., Wickersham T. A., Sawyer J. E., Miller R. K. et al. 2021. Maternal nutrient restriction in late pregnancy programs postnatal metabolism and pituitary development in beef heifers. PLoS One. 16(4):e0249924. 10.1371/journal.pone.0249924. 33831110 PMC8031383

[skaf455-B44] Love M. I. , Huber W., Anders S. 2014. Moderated estimation of fold change and dispersion for RNA-seq data with DESeq2. Genome Biol. 15(12):550. 10.1186/s13059-014-0550-8 25516281 PMC4302049

[skaf455-B45] McCarty K. J. , Owen M. P., Hart C. G., Thompson R. C., Burnett D. D., King E. H., Hopper R. M., Lemley C. O. 2018. Effect of chronic melatonin supplementation during mid to late getation on maternal uterine artery blood flow and subsequent development of male offspring in beef cattle. J. Anim. Sci. 96(12):5100–5111. 10.1093/jas/sky363. 30203092 PMC6276587

[skaf455-B46] McCoski S. , Bradbery A., Marques R. D. S., Posbergh C., Sanford C. 2021. Maternal nutrition and developmental programming of male progeny. Animals (Basel). 11(8):2216. 10.3390/ani11082216. 34438674 PMC8388505

[skaf455-B47] Melick C. H. , Meng D., Jewell J. L. 2020. A-kinase anchoring protein 8L interacts with mTORC1 and promotes cell growth. J. Biol. Chem. 295(23):8096–8105. 10.1074/jbc.AC120.012595. 32312749 PMC7278349

[skaf455-B48] Mezzacappa C. , Komiya Y., Habas R. 2012. Activation and function of small GTPases rho, rac, and Cdc42 during gastrulation. Methods Mol. Biol. 839:119–131. 10.1007/978-1-61779-510-7_10 22218897 PMC4414490

[skaf455-B49] Moore L. , Le T., Fan G. 2013. DNA methylation and its basic function. Neuropsychopharmacology. 38(1):23–38. 10.1038/npp.2012.112 22781841 PMC3521964

[skaf455-B50] Moujaber O , Stochaj U. 2020. The cytoskeleton as regulator of cell signaling pathways. Trends Biochem. Sci. 45(2):96–107. 10.1016/j.tibs.2019.11.003 31812462

[skaf455-B51] Namous H. , Peñagaricano F., Del Corvo M., Capra E., Thomas D. L., Stella A., Williams J. L., Marsan P. A., Khatib H. 2018. Integrative analysis of methylomic and transcriptomic data in fetal sheep muscle tissues in response to maternal diet during pregnancy. BMC Genomics. 19(1):123. 10.1186/s12864-018-4509-0. 29409445 PMC5801776

[skaf455-B52] Ng S. S. M. , Jorge S., Malik M., Britten J., Su S. C., Armstrong C. R., Brennan J. T., Chang S., Baig K. M., Driggers P. H. et al. 2019. A-Kinase anchoring protein 13 (AKAP13) augments progesterone signaling in uterine fibroid cells. J. Clin. Endocrinol. Metab. 104(3):970–980. 10.1210/jc.2018-01216. 30239831 PMC6365770

[skaf455-B53] NRC. 2000. Nutritional requirements of beef cattle. 7th rev. ed. Washington (DC): National Academies Press.

[skaf455-B54] Oliva R. 2006. Protamines and male infertility. Hum. Reprod. Update. 12(4):417–435. 10.1093/humupd/dml009 16581810

[skaf455-B55] Paradis F. , Wood K. M., Swanson K. C., Miller S. P., McBride B. W., Fitzsimmons C. 2017. Maternal nutrient restriction in mid-to-late gestation influences fetal mRNA expression in muscle tissues in beef cattle. BMC Genomics. 18(1):632. 10.1186/s12864-017-4051-5. 28821223 PMC5562975

[skaf455-B56] Pariante P. , Dotolo R., Venditti M., Ferrara D., Donizetti A., Aniello F., Minucci S. 2016. First evidence of DAAM1 localization during the post-natal development of rat testis and in mammalian sperm. J. Cell. Physiol. 231(10):2172–2184. 10.1002/jcp.25330 26831620

[skaf455-B57] Park E. , Kim G. H., Choi S. C., Han J. K. 2006. Role of PKA as a negative regulator of PCP signaling pathway during xenopus gastrulation movements. Dev. Biol. 292(2):344–357. 10.1016/j.ydbio.2006.01.011 16490187

[skaf455-B58] Park J. , Lee D. 2020. Functional roles of protein phosphatase 4 in multiple aspects of cellular physiology: a friend and a foe. BMB Rep. 53(4):181–190. 10.5483/BMBRep.2020.53.4.019 32192570 PMC7196183

[skaf455-B59] Patankar A. , Gajbhiye R., Surve S., Parte P. 2021. Epigenetic landscape of testis specific histone H2B variant and its influence on sperm function. Clin. Epigenetics. 13(1):101. 10.1186/s13148-021-01088-4. 33933143 PMC8088685

[skaf455-B60] Patel H. , Ewels P., Peltzer A., Hammarén R., Botvinnik O., Sturm G., Davenport C. 2020. nf-core/rnaseq: nf-core/rnaseq v3.0 - Silver Shark (Version 3.0). Zenodo.

[skaf455-B61] Pelizzola M , Ecker J. R. 2011. The DNA methylome. FEBS Lett. 585(13):1994–2000. 10.1016/j.febslet.2010.10.061 21056564 PMC3129437

[skaf455-B62] Peter W. H. , Ridwan A., Olanrewaju A., Andrey A., Matthieu G. A., If B., Arne B., Ruth B., Andrew B., Jyothish B. et al. 2024. Nucleic Acids Res. 52(D1):D891–D899. 10.1093/nar/gkad1049. 37953337 PMC10767893

[skaf455-B63] Picard Toolkit. 2019. Broad Institute, GitHub Repository. Broad Institute. http://broadinstitute.github.io/picard/

[skaf455-B64] Polizel G. H. G. , Strefezzi R. D. F., Cracco R. C., Fernandes A. C., Zuca C. B., Castellar H. H., Baldin G. C., Santana M. H. D. A. 2021. Effects of different maternal nutrition approaches on weight gain and on adipose and muscle tissue development of young bulls in the rearing phase. Trop. Anim. Health Prod. 53(6):536. 10.1007/s11250-021-02982-y. 34751823

[skaf455-B65] Qian M. X. , Pang Y., Liu C. H., Haratake K., Du B. Y., Ji D. Y., Wang G. F., Zhu Q. Q., Song W., Yu Y. et al. 2013. Acetylation-mediated proteasomal degradation of core histones during DNA repair and spermatogenesis. Cell. 153(5):1012–1024. 10.1016/j.cell.2013.04.032 23706739 PMC3983474

[skaf455-B66] Qu J. , Sun M., Wang X., Song X., He H., Huan Y. 2020. Melatonin enhances the development of porcine cloned embryos by improving DNA methylation reprogramming. Cell. Reprogram. 22(3):156–166. 10.1089/cell.2019.0103 32207988

[skaf455-B67] Quinlan A. R. , Hall I. R. 2010. BEDTools: a flexible suite of utilities for comparing genomic features. Bioinform. 26(6):841–842. 10.1093/bioinformatics/btq033 PMC2832824 20110278

[skaf455-B68] Raudvere U. , Kolberg L., Kuzmin I., Arak T., Adler P., Peterson H., Vilo J. 2019. g: Profiler: a web server for functional enrichment analysis and conversions of gene lists (2019 update). Nucleic Acids Res. 47(W1):W191–W198. 10.1093/nar/gkz369 31066453 PMC6602461

[skaf455-B69] Reiter R. J. , Tan D. X., Gitto E. et al. 2004. Pharmacological utility of melatonin in reducing oxidative cellular and molecular dam- age. Pol. J. Pharmacol. 56:159–170. PMID:15156066. https://if-pan.krakow.pl/pjp/pdf/2004/2_159.pdf 15156066

[skaf455-B70] Reynolds L. P. , Redmer D. A. 1995. Utero-placental vascular development and placental function. J. Anim. Sci. 73(6):1839–1851. 10.2527/1995.7361839x 7545661

[skaf455-B71] Reynolds L. P. , Borowicz P. P., Caton J. S., Crouse M. S., Dahlen C. R., Ward A. K. 2019. Developmental programming of fetal growth and development. Vet. Clin. North Am. Food Anim. Pract. 35(2):229–247. 10.1016/j.cvfa.2019.02.006. 31103178

[skaf455-B72] Rossitto M. , Déjardin S., Rands C. M., Le Gras S., Migale R., Rafiee M.-R., Neirijnck Y., Pruvost A., Nguyen A. L., Bossis G. et al. 2022. TRIM28-dependent SUMOylation protects the adult ovary from activation of the testicular pathway. Nat. Commun. 13(1):4412. 10.1038/s41467-022-32061-1. 35906245 PMC9338040

[skaf455-B73] Sayols S. , Scherzinger D., Klein H. 2016. dupRadar: a bioconductor package for the assessment of PCR artifacts in RNA-Seq data. BMC Bioinform. 17(1):428. 10.1186/s12859-016-1276-2 PMC5073875 27769170

[skaf455-B74] Siomi M. C. , Mannen T., Siomi H. 2010. How does the royal family of tudor rule the PIWI-interacting RNA pathway? Genes Dev. 24(7):636–646. 10.1101/gad.1899210 20360382 PMC2849120

[skaf455-B75] Skobowiat C. , Broz ˙yna A. A., Janjetovic Z., Jeayeng S., Oak A. S. W., Kim T. K., Panich U., Reiter R. J., Slominski A. T. 2018. Melatonin and its derivatives counteract the ultraviolet B Radiation-Induced damage in human and porcine skin ex vivo. J. Pineal Res. 65:e12501. 10.1111/jpi.12501 29702749 PMC6105533

[skaf455-B76] Slominski A. T. , Semak I., Fischer T. W., Kim T.-K., Kleszczyński K., Hardeland R., Reiter R. J. 2017. Metabolism of melatonin in the skin: Why is it important? Exp. Dermatol. 26(7):563–568. 10.1111/exd.13208 27619234 PMC5348284

[skaf455-B77] Suzuki M. M. , Bird A. 2008. DNA methylation landscapes: provocative insights from epigenomics. Nat. Rev. Genet. 9(6):465–476. 10.1038/nrg2341 18463664

[skaf455-B78] Tang H. , Brennan J., Karl J., Hamada Y., Raetzman L., Capel B. 2008. Notch signaling maintains leydig progenitor cells in the mouse testis. Development. 135(22):3745–3753. 10.1242/dev.024786. 18927153 PMC3653410

[skaf455-B79] Veeman M. T. , Axelrod J. D., Moon R. T. 2003. A second canon. Functions and mechanisms of beta-catenin-independent wnt ­signaling. Dev. Cell. 5(3):367–377. 10.1016/s1534-5807(03)00266-1 12967557

[skaf455-B80] Venditti M. , Ben Rhouma M., Romano M. Z., Messaoudi I., Reiter R. J., Minucci S. 2021. Altered expression of *DAAM1* and PREP induced by cadmium toxicity is counteracted by melatonin in the rat testis. Genes. (Basel). 12(7):1016. 10.3390/genes12071016. 34208970 PMC8304460

[skaf455-B81] Venditti M. , Santillo A., Falvo S., Fiore M. M. D., Baccari G. C., Minucci S. 2020. D-Aspartate upregulates DAAM1 protein levels in the rat testis and induces its localization in spermatogonia nucleus. Biomolecules. 10(5):677. 10.3390/biom10050677. 32353957 PMC7277804

[skaf455-B82] Venny O. J. C. An Interactive Tool for Comparing Lists with Venn Diagrams. 2007. [accessed November 18, 2025]. http://bioinfogp.cnb.csic.es/tools/venny/index.html.

[skaf455-B83] Vogl W. , Lyon K., Adams A., Piva M., Nassour V. 2018. The endoplasmic reticulum, calcium signaling and junction turnover in sertoli cells. Reproduction. 155(2):R93–R104. 10.1530/REP-17-0281 29066527

[skaf455-B84] Vonnahme K. A. , Lemley C. O. 2011. Programming the offspring through altered uteroplacental hemodynamics: how maternal environment impacts uterine and umbilical blood flow in cattle, sheep and pigs. Reprod. Fertil. Dev. 24(1):97–104. 10.1071/RD11910 22394721

[skaf455-B85] Wallingford J. B. , Fraser S. E., Harland R. M. 2002. Convergent extension: the molecular control of polarized cell movement during embryonic development. Dev. Cell. 2(6):695–706. 10.1016/s1534-5807(02)00197-1 12062082

[skaf455-B86] Wang L. , Wang S., Li W. 2012. RSeQC: quality control of RNA-seq experiments. Bioinformatics. 28(16):2184–2185. 10.1093/bioinformatics/bts356 22743226

[skaf455-B87] Wang Q. , Zhang M., Ding Y., Wang Q., Zhang W., Song P., Zou M. H. 2014. Activation of NAD(P)H oxidase by tryptophan-derived 3-hydroxykynurenine accelerates endothelial apoptosis and dysfunction in vivo. Circ. Res. 114(3):480–492. 10.1161/CIRCRESAHA.114.302113 24281189 PMC4104160

[skaf455-B88] Xu K. , Liu G., Fu C. 2018. The tryptophan pathway targeting antioxidant capacity in the placenta. Oxid. Med. Cell. Longev. 2018:1054797. 10.1155/2018/1054797 30140360 PMC6081554

[skaf455-B89] Yan X. , Zhu M. J., Dodson M. V., Du M. 2013. Developmental programming of fetal skeletal muscle and adipose tissue development. J. Genomics. 1:29–38. 10.7150/jgen.3930 25031653 PMC4091428

[skaf455-B90] Yang W. C. , Tang K. Q., Fu C. Z., Riaz H., Zhang Q., Zan L. S. 2014. Melatonin regulates the development and function of bovine sertoli cells via its receptors MT1 and MT2. Anim. Reprod. Sci. 147(1-2):10–16. 10.1016/j.anireprosci.2014.03.017. 24768045

[skaf455-B91] Zhang Y. , Otomaru K., Oshima K., Goto Y., Oshima I., Muroya S., Sano M., Saneshima R., Nagao Y., Kinoshita A. et al. 2021. Effects of low and high levels of maternal nutrition consumed for the entirety of gestation on the development of muscle, adipose tissue, bone, and the organs of wagyu cattle fetuses. Anim. Sci. J. 92(1):e13600. 10.1111/asj.13600. 34327770 PMC9285072

