# Supplementary material for: Integrated fetal testicular transcriptomic and epigenomic profiles during maternal nutrient restriction with dietary melatonin intervention
Source: J Anim Sci. 2026 Jan 9;104:skaf455. doi: 10.1093/jas/skaf455 (PMC12863962; doi:10.1093/jas/skaf455)
Supplement: skaf455_Supplementary_Data [file skaf455_supplementary_data.zip › Supplemental Figures.docx]

**Supplemental Figure S1: (A)** Distribution of methylation values per sample, **(B)** Distribution of methylation values per group, adequately fed (ADQ-CON; 100% NRC recommendation, S9957, S6223, S5392), nutrient restricted (RES-CON, 60% NRC recommendation, S4591, S9964, S676, S9869, S865), and adequately fed or nutrient restricted supplemented with 20 mg/d of melatonin (ADQ-MEL, S8556, S5741, S9240, S9738, S8961; RES-MEL, S6721, S6768, S7059, S9866)


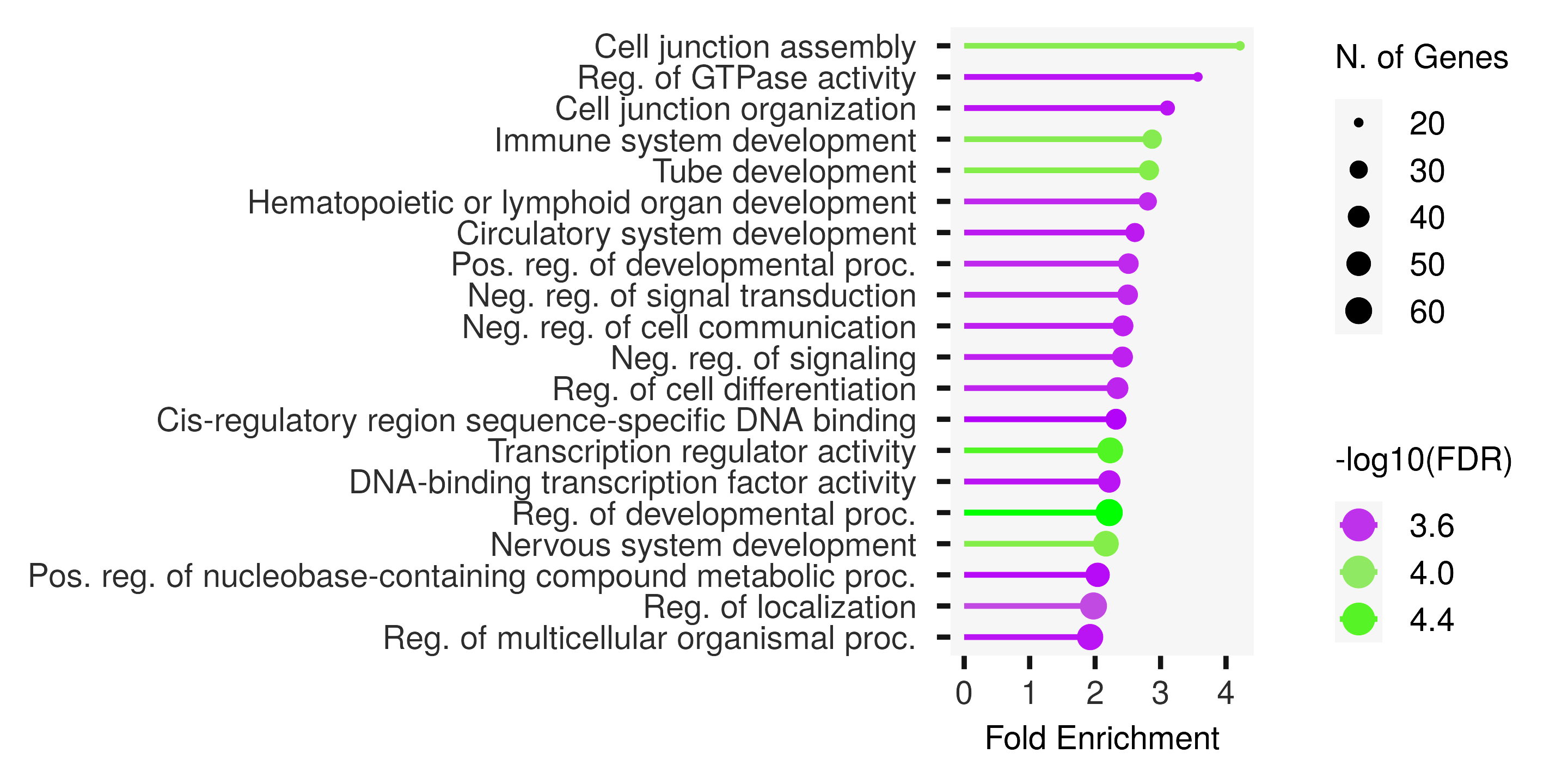


**Supplemental Figure S2:** Summary of enriched Gene Ontology (GO) components in RES-CON vs ADQ-CON fetal testicular tissue. The dot plot represents results based on the differentially methylated genes (DMGs) in enrichment in the ontology terms. The color represents statistical significance. The Y-axis corresponds to enriched GO terms and the X-axis represents fold enrichment (the proportion of differentially methylated genes (DMGs) vs. all the genes annotated with GO terms. The size of the dot represents the number of genes annotated to GO terms and the color represents the -log10(FDR).


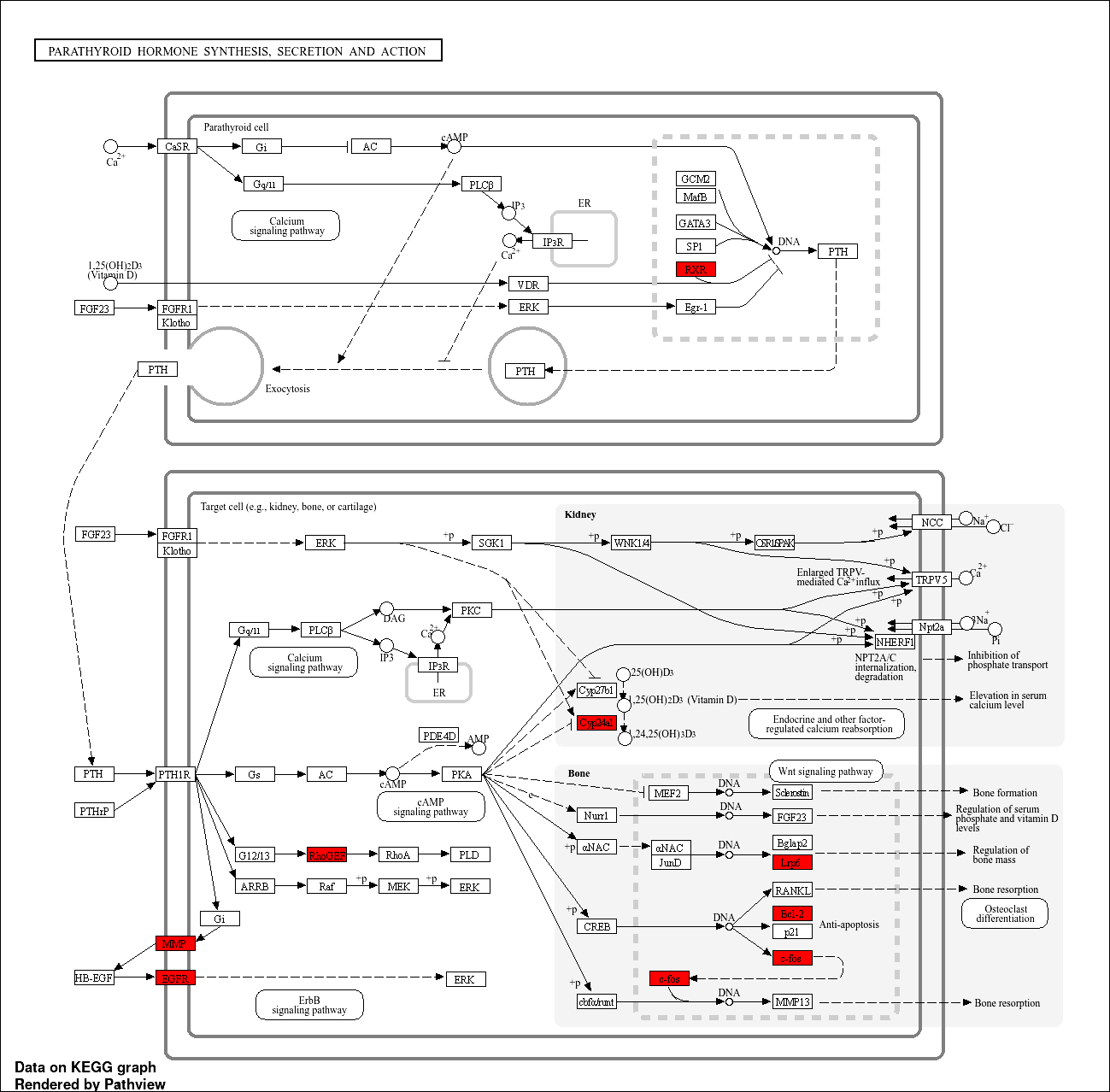


**Supplemental Figure S3:** Parathyroid hormone synthesis, secrection and action pathway with some differentlly methylated genes (Red boxes) which involved in this pathway (RES-CON vs ADQ-CON fetal testicular tissue). Data on KEGG graph rendered by pathview.


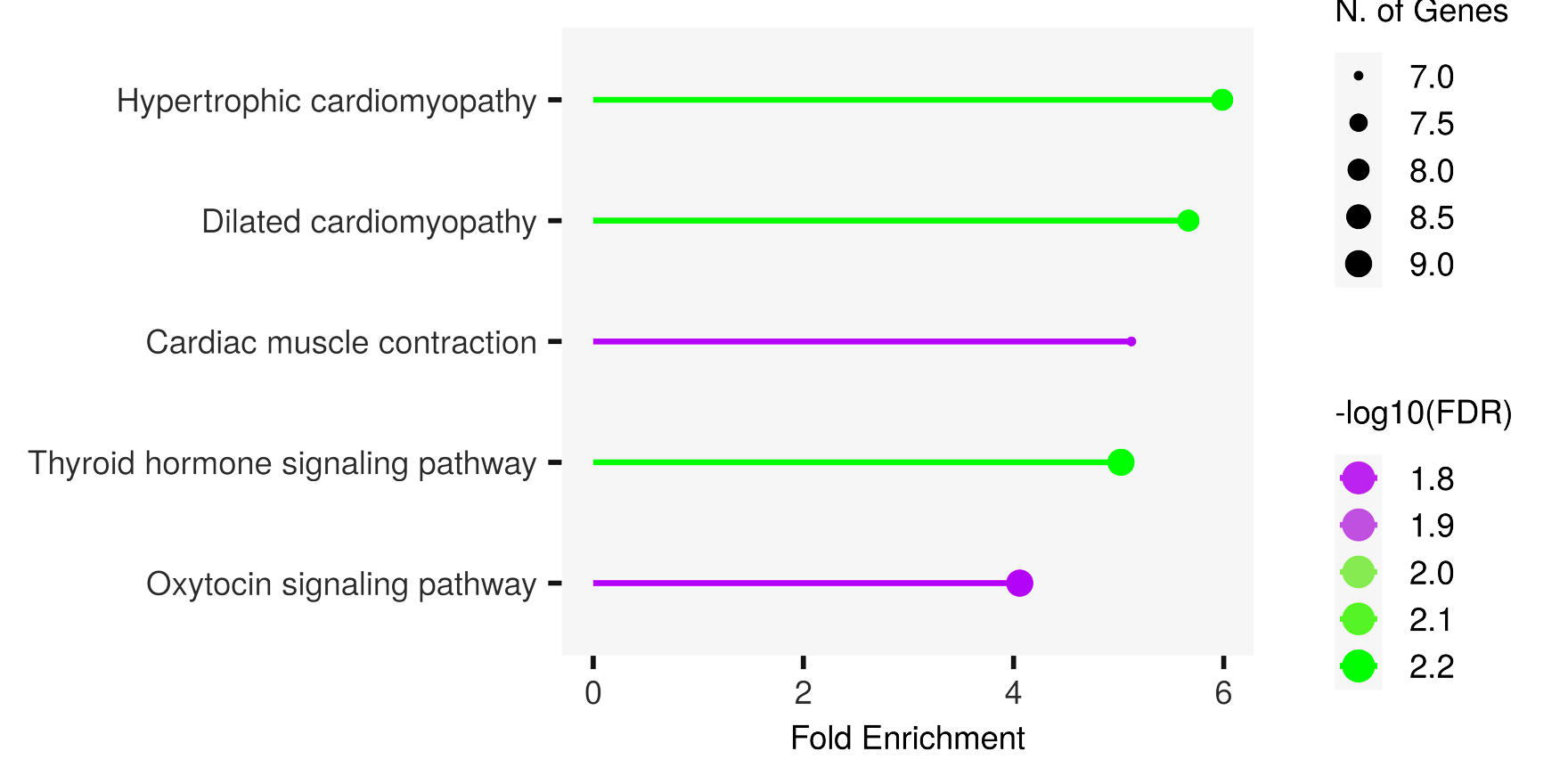


**Supplemental Figure S4:** Summary of enriched Gene Ontology (GO) components in ADQ-MEL vs ADQ-CON fetal testicular tissue. The dot plot represents results based on the differentially methylated genes (DMGs) in enrichment in the ontology terms. The color represents statistical significance. The Y-axis corresponds to enriched GO terms and the X-axis represents fold enrichment (the proportion of differentially methylated genes (DMGs) vs. all the genes annotated with GO terms. The size of the dot represents the number of genes annotated to GO terms and the color represents the -log10(FDR).


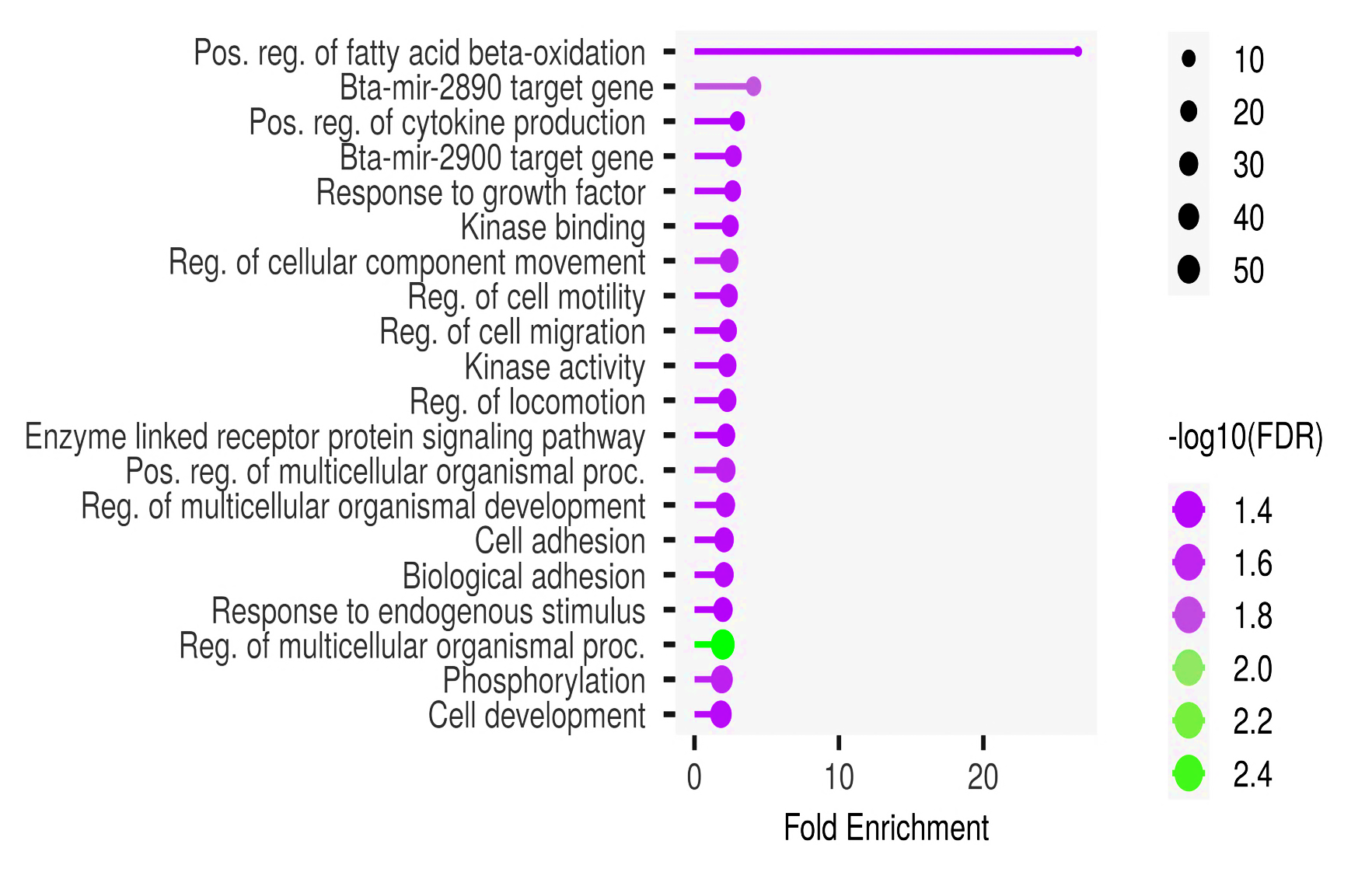


**Supplemental Figure S5:** Summary of enriched Gene Ontology (GO) components in RES-MEL vs RES-CON fetal testicular tissue. The dot plot represents results based on the differentially methylated genes (DMGs) in enrichment in the ontology terms. The color represents statistical significance. The Y-axis corresponds to enriched GO terms and the X-axis represents fold enrichment (the proportion of differentially methylated genes (DMGs) vs. all the genes annotated with GO terms. The size of the dot represents the number of genes annotated to GO terms and the color represents the -log10(FDR).


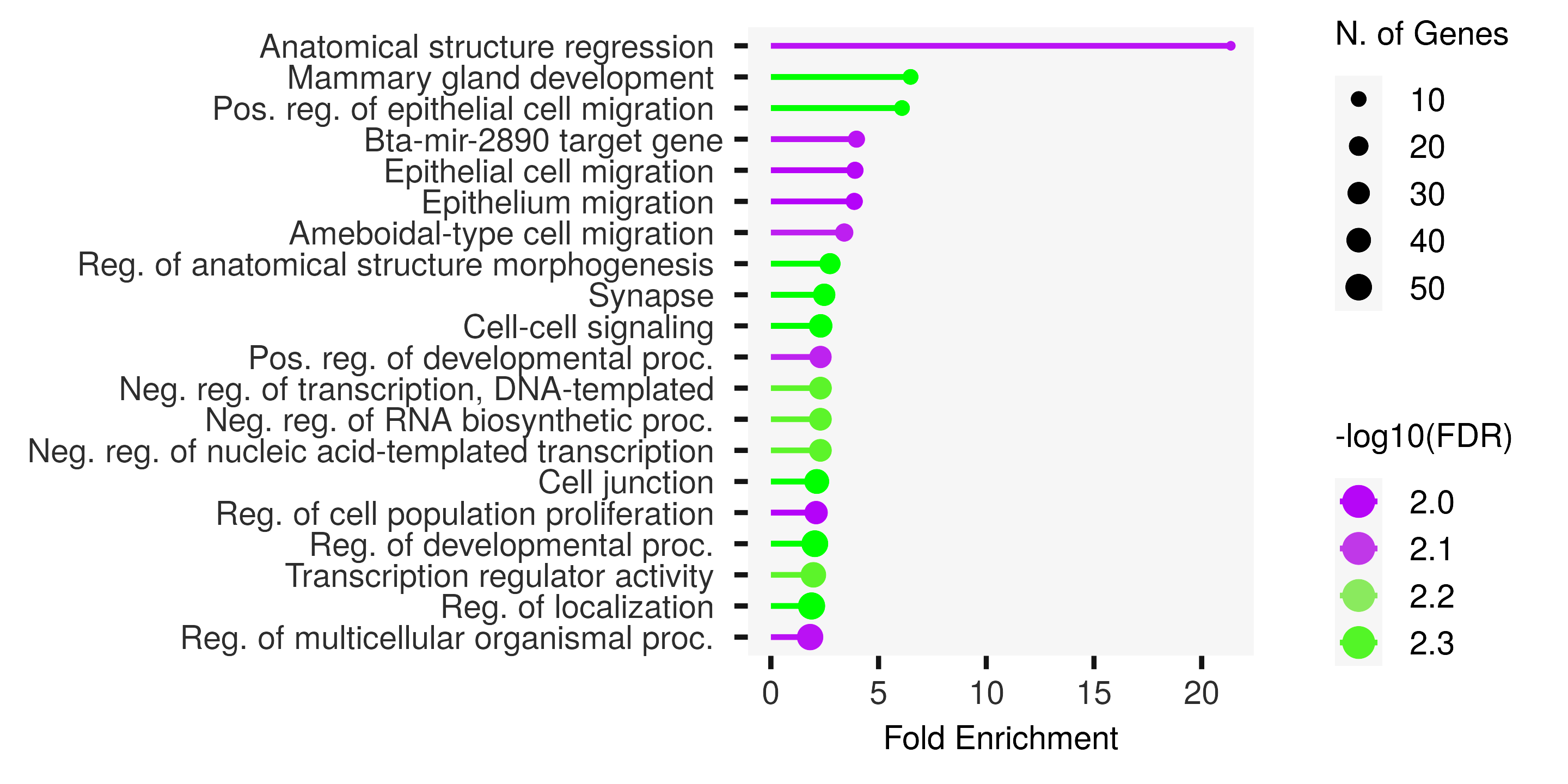


**Supplemental Figure S6:** Summary of enriched Gene Ontology (GO) components in RES-MEL vs ADQ-MEL fetal testicular tissue. The dot plot represents results based on the differentially methylated genes (DMGs) in enrichment in the ontology terms. The color represents statistical significance. The Y-axis corresponds to enriched GO terms and the X-axis represents fold enrichment (the proportion of differentially methylated genes (DMGs) vs. all the genes annotated with GO terms. The size of the dot represents the number of genes annotated to GO terms and the color represents the -log10(FDR).
